# Comparison of the efficacy of hematopoietic stem cell mobilization regimens: a systematic review and network meta-analysis of preclinical studies

**DOI:** 10.1186/s13287-021-02379-6

**Published:** 2021-05-29

**Authors:** Chengxin Luo, Li Wang, Guixian Wu, Xiangtao Huang, Yali Zhang, Yanni Ma, Mingling Xie, Yanni Sun, Yarui Huang, Zhen Huang, Qiuyue Song, Hui Li, Yu Hou, Xi Li, Shuangnian Xu, Jieping Chen

**Affiliations:** 1 Center for Hematology, Southwest Hospital, Third Military Medical University, #30 Gaotanyan Street, Shapingba District, Chongqing, 400038 China; 2 Key Laboratory of Cancer Immunotherapy of Chongqing, Chongqing, China; 3 Department of Hematology, The First Affiliated Hospital of Chongqing Medical University, Chongqing, China; 4 Department of Health Statistics, Third Military Medical University, Chongqing, China; 5 Institute of Infectious Disease, Southwest Hospital, Third Military Medical University, #30 Gaotanyan Street, Shapingba District, Chongqing, 400038 China

**Keywords:** Hematopoietic stem cells, Mobilization, Animal studies, Network meta-analysis

## Abstract

**Background:**

Mobilization failure may occur when the conventional hematopoietic stem cells (HSCs) mobilization agent granulocyte colony-stimulating factor (G-CSF) is used alone, new regimens were developed to improve mobilization efficacy. Multiple studies have been performed to investigate the efficacy of these regimens via animal models, but the results are inconsistent. We aim to compare the efficacy of different HSC mobilization regimens and identify new promising regimens with a network meta-analysis of preclinical studies.

**Methods:**

We searched Medline and Embase databases for the eligible animal studies that compared the efficacy of different HSC mobilization regimens. Primary outcome is the number of total colony-forming cells (CFCs) in per milliliter of peripheral blood (/ml PB), and the secondary outcome is the number of Lin^−^ Sca1^+^ Kit^+^ (LSK) cells/ml PB. Bayesian network meta-analyses were performed following the guidelines of the National Institute for Health and Care Excellence Decision Support Unit (NICE DSU) with WinBUGS version 1.4.3. G-CSF-based regimens were classified into the SD (standard dose, 200–250 μg/kg/day) group and the LD (low dose, 100–150 μg/kg/day) group based on doses, and were classified into the short-term (2–3 days) group and the long-term (4–5 days) group based on administration duration. Long-term SD G-CSF was chosen as the reference treatment. Results are presented as the mean differences (MD) with the associated 95% credibility interval (95% CrI) for each regimen.

**Results:**

We included 95 eligible studies and reviewed the efficacy of 94 mobilization agents. Then 21 studies using the poor mobilizer mice model (C57BL/6 mice) to investigate the efficacy of different mobilization regimens were included for network meta-analysis. Network meta-analyses indicated that compared with long-term SD G-CSF alone, 14 regimens including long-term SD G-CSF + Me6, long-term SD G-CSF + AMD3100 + EP80031, long-term SD G-CSF + AMD3100 + FG-4497, long-term SD G-CSF + ML141, long-term SD G-CSF + desipramine, AMD3100 + meloxicam, long-term SD G-CSF + reboxetine, AMD3100 + VPC01091, long-term SD G-CSF + FG-4497, Me6, long-term SD G-CSF + EP80031, POL5551, long-term SD G-CSF + AMD3100, AMD1300 + EP80031 and long-term LD G-CSF + meloxicam significantly increased the collections of total CFCs. G-CSF + Me6 ranked first among these regimens in consideration of the number of harvested CFCs/ml PB (MD 2168.0, 95% CrI 2062.0−2272.0). In addition, 7 regimens including long-term SD G-CSF + AMD3100, AMD3100 + EP80031, long-term SD G-CSF + EP80031, short-term SD G-CSF + AMD3100 + IL-33, long-term SD G-CSF + ML141, short-term LD G-CSF + ARL67156, and long-term LD G-CSF + meloxicam significantly increased the collections of LSK cells compared with G-CSF alone. Long-term SD G-CSF + AMD3100 ranked first among these regimens in consideration of the number of harvested LSK cells/ml PB (MD 2577.0, 95% CrI 2422.0–2733.0).

**Conclusions:**

Considering the number of CFC and LSK cells in PB as outcomes, G-CSF plus AMD3100, Me6, EP80031, ML141, FG-4497, IL-33, ARL67156, meloxicam, desipramine, and reboxetine are all promising mobilizing regimens for future investigation.

**Supplementary Information:**

The online version contains supplementary material available at 10.1186/s13287-021-02379-6.

## Background

Hematopoietic stem cell transplantation (HSCT) is a life-saving strategy for variety of severe disorders, including bone marrow failure after high-dose radiation and various hematological malignancies [1]. Peripheral blood stem cells (PBSCs) have been gradually replaced bone marrow (BM) as the predominant source of stem cell for transplantation in clinical practice [2, 3]. It has been demonstrated that PBSCs transplantation is associated with more convenient and safer harvest procedure, faster hematological recovery, lower risk of graft failure, and comparable disease-free survival (DFS) and overall survival (OS) in comparison with BM transplantation [4–6]. However, hematopoietic stem cells (HSCs) mainly reside in specialized BM niches during steady state; the number of HSCs circulating in peripheral blood (PB) is very low and not sufficient for harvest [6]. Administration of exogenous cytokines or chemokines could induce the egress of HSCs from BM into PB in a process termed mobilization. Successful mobilization allows for efficient collection of HSCs sufficient for transplantation, and increment in the dose of harvested HSCs could improve transplantation efficiency via promoting hematopoietic reconstitution, as well as reducing the need for supportive blood transfusion and the risks of infections [7, 8]. Therefore, efficient mobilization is the key to successful HSCT and sustained hematopoietic recovery.

Granulocyte colony-stimulating factor (G-CSF) is the most commonly used steady-state HSC mobilization agent in clinical practice. However, mobilization failure may occur when G-CSF is used alone [8]. In addition, mobilization using G-CSF alone requires multiple doses beginning at least 4 days before first apheresis and a median of 2–5 apheresis sessions to collect sufficient PBSCs, which increased the risk of adverse events [7]. The incidences of bone pain induced by G-CSF is higher than 80% at day 4, in addition, other G-CSF-related severe adverse events including myocardial infarctions, pulmonary embolism, and splenic rupture also have been reported [9–11]. To improve mobilization efficacy and attenuate toxicity, novel mobilization regimens are developed and investigated in a variety of animal studies before applied in clinical practice, but the results are inconsistent. This study aims to review and compare the efficacy of different HSC mobilization regimens and identify new promising regimens with a network meta-analysis of preclinical studies, which may be helpful for guiding future clinical trials.

## Methods

### Literature search and study selection

We searched Medline and Embase from inception to February 23, 2021, with the search term “stem cell mobilization” and a filter of “animals”. The titles and abstracts of retrieved citations were independently screened by two investigators (CXL and XL) for eligibility. Disagreements were resolved by full-text review and discussion with a third investigator (SNX). Preclinical studies that met the following criteria were included for review: (1) compared the efficacy of two or more different regimens in the mobilization of hematopoietic stem and progenitor cells (HSPCs) and (2) using any species of mice as experimental animals. As for network meta-analysis, the inclusion criteria were (1) using the poor mobilizer mice model-C57BL/6 mice as experimental animals [12] and (2) reporting data for at least one of the outcomes of mobilization efficacy, including the number of total colony-forming cells (CFCs) and Lin^−^ Sca1^+^ Kit^+^ (LSK) cells per milliliter of peripheral blood (/ml PB). Since aged mice were reported to have better mobilization efficiency compared with young mice and no significant difference was reported among mice younger than 3 months, we excluded studies using mice older than 12 weeks in meta-analysis to reduce heterogeneity [13]. In addition, we only included studies that administrated G-CSF via subcutaneously injection. Furthermore, G-CSF-based regimens were classified into the SD (standard dose, 200–250 μg/kg/day) group and the LD (low dose, 100–150 μg/kg/day) group based on G-CSF doses and were classified into the short-term (2–3 days) group and the long-term (4–5 days) group based on administration duration of G-CSF. Studies with significant heterogeneity in dosage and injection route of G-CSF were excluded in meta-analysis.

### Data extraction and quality assessment

Full text of all eligible studies was reviewed, and two investigators (CXL and XL) independently extracted data using predesigned data collection forms. Data was extracted on studies characteristics, animal’s characteristics, dosage of mobilization regimens, and efficacy outcomes. We chose the number of total CFCs per milliliter PB as primary outcome, and the number of LSK cells per milliliter PB as secondary outcome. The mean, standard deviation (SD) or standard error (SE) of each outcome are extracted directly from published text or from related graphs with Adobe Photoshop version CS3 via previously validated methods [14]. The methodological quality of included studies was assessed using the SYstematic Review Centre for Laboratory animal Experimentation (SYRCLE) risk of bias tool, which contains 10 items, including random sequence generation, similar baseline characteristics, allocation concealment, random housing, blinding of caregivers and investigators, random selection for outcome assessment, blinding of outcome assessor, adequate addressing of incomplete outcome data, free from selective outcome reporting, and free from other bias [15]. For each item, judgment of “yes”, “no”, and “unclear” respectively indicate low, high and unclear risk of bias.

### Statistical analyses

We conducted network meta-analyses to compare the efficacy of multiple mobilization regimens simultaneously. Network plot for each outcome was obtained using Stata version 12.0. Bayesian network meta-analyses were performed with WinBUGS version 1.4.3 (MRC Biostatistics Unit, Cambridge, UK), employing the Markov Chain Monte Carlo (MCMC) approach and following the guidelines of the National Institute for Health and Care Excellence Decision Support Unit (NICE DSU) [16]. We used the WinBUGS code previously established by Dias et al*.*, which could handle trials with multiple arms and rank treatments with additional code [16]. Three chains were run to yield 150,000 iterations, and the initial 5000 burn-ins were discarded. The convergence of models was assessed with trace plots and Brooks-Gelman-Rubin statistic. Model fit of fixed-effect model and random-effect model were compared with the Deviance Information Criterion (DIC), and model with lower DIC was adopted. Long-term SD G-CSF monotherapy was chosen as the common comparator. Estimates of treatment effects were reported as mean differences (MD) with the associated 95% credibility interval (95% CrI). The 95% CrI calculated in Bayesian meta-analysis can be interpreted like the 95% confidence intervals (95% CI) in traditional meta-analysis [17]. The probability of each regimen to be the best was calculated by ranking the relative effects of all treatments in each iteration and defined as the proportion of times a regimen ranked first. This work is reported according to the Preferred Reporting Items for Systematic Reviews and Meta-analyses (PRISMA) extension statement for network meta-analyses [18].

## Results

### Characteristics of included trials

**Fig. 1:**
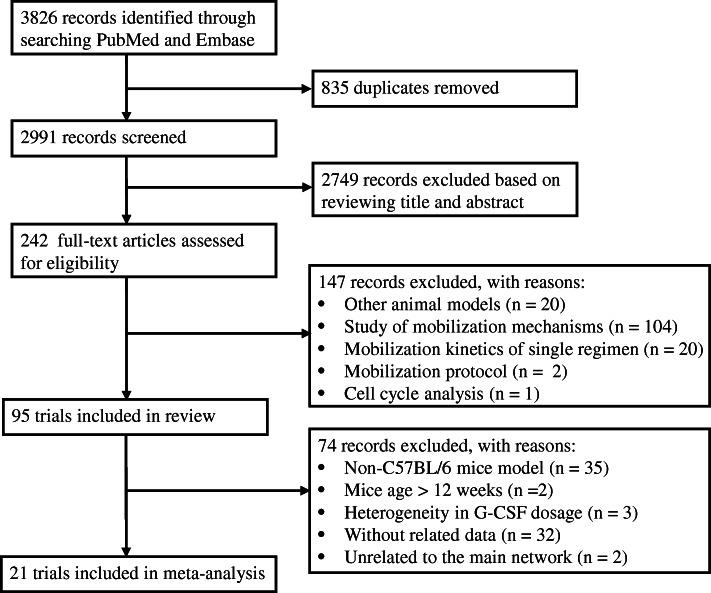
Flow chart of study selection. The PRISMA flow chart of study screening and selection

We identified 3826 records from database searches. After removing 835 duplicates, 2991 records were screened on title and abstract, and 2749 clearly irrelevant records were excluded. We retrieved the full text of the remaining 242 records for further assessment. We excluded 147 records for the reasons listed in the flow diagram (Fig. 1). Ultimately, 95 eligible studies were included for review [19–113]. The characteristics of the 95 included studies are summarized in Supplementary Table 1. The efficacy of 94 HSC mobilization agents were investigated, including cytokines, agents targeting the CXCR4 (C-X-C chemokine receptor type 4)/CXCL12 (SDF1, stromal cell-derived factor-1) axis, agents targeting the VLA-4(very late antigen-4)/VCAM-1 (vascular cell adhesion molecule-1) axis, chemotherapeutic agents, nonsteroidal anti-inflammatory drugs (NSAIDs), and other agents. Most of these agents not only can induce the mobilization of hematopoietic stem and progenitor cells (HSPCs) alone, but also can enhanced the mobilization mediated by G-CSF or AMD3100 synergistically or additively. The detailed information and mobilization efficacy of these agents are reviewed in Table 1. Compared with the conventional G-CSF, modified G-CSF including SD/0 (an engineered pegylated G-CSF), IMG-CSF (G-CSF immobilized on polyethylenoxide by nanotechnology), and PEGLip-G-CSF (pegylated liposome formulated G-CSF) exhibited enhanced mobilization efficacy. Among other cytokines, IL-33 showed superior mobilization potential than G-CSF and AMD3100, and tGROβ (a truncated form of chemokine GROβ) showed superior mobilization AMD3100. AMD3100-a CXCR4 antagonist was the most commonly used agents in combined regimens, which can significantly increase mobilization with a single dose when in combination with G-CSF. There are 7 new CXCR4 antagonist investigated and compared with AMD 3100, among which T-140, POL5551, and CX0714 showed significant superior mobilization AMD3100.

**Table 1:** Detailed information and mobilization efficacy of all included novel agents

Agent	Detailed information	HSPC mobilization efficacy	References
**1. Cytokines**			
SCF	Stem cell factor	Multi-day SCF alone can induce mobilization; IL-11 plus SCF synergistically enhanced mobilization.	Mauch et al. [21]
IL-11	Interleukin-11	Multi-day IL-11 alone can induce mobilization; IL-11 plus SCF or G-CSF synergistically enhanced mobilization.	Mauch et al. [21], Meng et al. [42]
MIP-1α	Macrophage inflammatory protein 1α	Single-dose MIP-1α alone induced rapid mobilization; MIP-1α markedly enhanced G-CSF- and AMD3100-induced mobilization.	Lord et al. [20], Broxmeyer et al. [52]
IL-8	Interleukin-8	Single-dose IL-8 alone induced rapid mobilization; the combination of IL-8 with G-CSF or CWHM-823 enhanced mobilization.	Wang et al. [24], Zhang et al. [28], de Kruijf et al. [66], Karpova et al. [107]
MIP-2	Macrophage inflammatory protein-2	Single-dose MIP-2 alone induced rapid mobilization; MIP-2 markedly enhanced G-CSF-induced mobilization.	Wang et al. [24]
FLT-3L	Fms-like tyrosine kinase-3 ligand	Multi-dose FLT-3L alone can induce mobilization; FLT-3L synergistically enhanced mobilization induced by G-CSF, GM-CSF, IL-8, and AMD 3100.	Brasel et al. [22], Sudo et al. [23], Neipp et al. [25], Robinson et al. [33], Robinson et al. [44], de Kruijf et al. [66], He et al. [83]
GM-CSF	Granulocyte macrophage colony-stimulating factor	GM-CSF alone did not induce significant mobilization; FLT-3L + GM-CSF synergistically enhanced mobilization.	Brasel et al. [22]; Robinson et al. [33]
PEG-MGDF	Pegylated megakaryocyte growth and development factor	Multi-day PEG-MGDF alone can induce mobilization; PEG-MGDF synergizes with G-CSF to enhance mobilization.	Torii et al. [27], Honda et al. [36]
SD/01^a^, IM G-CSF^a^, and PEGLip-G-CSF^a^	Modified G-CSF	Modified G-CSF exhibited superior mobilization potential compared with standard G-CSF	De Haan et al. [30], Dygai et al. [62], Yatuv et al. [65]
GROβ and tGROβ^b^	GROβ: CXCL2, a chemokine; tGROβ: truncated form of GROβ (also known as SB-251353, GROβ_T_, GROβ_△4_)	Single-dose GROβ or tGROβ alone induced rapid mobilization; tGRO-β synergistically enhanced the mobilization effects of G-CSF, AMD3100, and VLA-4 antagonists.	King et al. [37], Pelus et al. [47], Fukuda et al et al. [54], Hoggatt et al. [77], Karpova et al. [107]
rhPlGF-1	Recombinant human placental growth factor-1	rhPlGF-1 alone had no mobilizing activity; rhPlGF-1 synergized with G-CSF in mobilization.	Carlo-Stella et al. [53]
LECT2	Leukocyte cell-derived chemotaxin 2	5-day LECT2 alone induced mobilization; the combination of LECT2 synergistically enhanced AMD3100 — but not G-CSF-induced mobilization.	Lu et al. [91]
GROα	The CXCR2 ligand CXCL1	Single-dose GROα alone induced rapid mobilization, the combination of GROα with CWHM-823 enhanced mobilization.	Karpova et al. [107]
IL-33^ab^	Interleukin-33	3-day IL-33 alone mobilized HSPCs more efficiently than G-CSF or AMD3100; IL-33 additively enhanced G-CSF- and AMD 3100-induced mobilization.	Alt et al. [105]
CSF1-Fc	CSF1 Fc fusion protein	CSF1-Fc enhanced G-CSF-induced mobilization.	Kaur et al. [113]
**2. Agents targeting CXCR4/CXCL12 (SDF1) axis**			
**2.1 CXCR4 antagonists**			
AMD3100	Plerixafor, a CXCR4 antagonist	Single-dose AMD 3100 alone can induce rapid mobilization; AMD 3100 synergizes with G-CSF to mobilize HSPCs.	Broxmeyer et al. [48]; Abraham et al. [51], Bonig et al. [61]
T-140^b^	4F-benzoyl-TN14003, a highly selective CXCR4 antagonist	T-140 has superior mobilization potential than AMD 3100; T-140 synergizes with G-CSF to mobilize HSPCs with higher efficacy than G-CSF + AMD 3100.	Abraham et al. [51]
TG-0054	A novel CXCR4 antagonist	Single-dose TG-0054 alone can induce mobilization; TG-0054 showed synergistic effects when combined with G-CSF.	Huang et al. [63]
POL5551^b^	A novel peptidic CXCR4 antagonist	Single-dose POL5551 induced higher levels of mobilization than AMD 3100; POL5551 synergizes with G-CSF and CY in mobilization; continuous infusion of POL5551 for 1–2 weeks achieved higher mobilization than G-CSF.	Karpova et al. [78], Karpova et al. [94]
ALT1188	A small molecule CXCR4 antagonist	Single-dose ALT1188 alone can induce rapid mobilization; Continuous infusion of ALT1188 for 2 weeks achieved higher mobilization than G-CSF.	Karpova et al. [94]
KRH3955	A chemically distinct CXCR4 antagonist	Single-dose KRH3955 alone induced rapid mobilization; the combination of KRH3955 with AMD 3100 did not enhance mobilization.	Redpath et al. [97]
CX0714^b^	A selective and potent CXCR4 antagonist	CX0714 has greater mobilization ability than AMD 3100; CX0714 synergistically enhanced G-CSF-induced mobilization with higher efficacy than G-CSF + AMD3100.	Wu et al. [103]
HF51116	A small molecule antagonist of CXCR4	The mobilization efficacy of HF51116 was comparable to AMD 3100; HF51116 synergistically enhanced G-CSF-induced mobilization.	Fang et al. [112]
**2.2 Other agents targeting CXCR4/CXCL12 axis**			
CTCE-0021	An SDF-1 analog	Single-dose CTCE-0021 alone can induce rapid mobilization; CTCE-0021 synergizes with G-CSF in mobilization.	Pelus et al. [49]
SCA	Sulfated colominic acid, a compound that can modulate CXCR4 function	Single-dose SCA alone can induce rapid mobilization; SCA synergizes with G-CSF in mobilization	Kubonishi et al. [56]
ATI-2341	A pepducin CXCR4 agonist	Single-dose ATI-2341 alone induced mobilization with similar efficacy to AMD3100	Tchernychev et al. [68]
APACs (Neo-r9, Neam-r9) and r9	Compounds that can compete with CXCL12 binding to CXCR4	Neo-r9, Neam-r9, and r9 induced robust mobilization similar to AMD3100 when used alone and showed additive effects when combined with AMD3100.	Berchanski et al. [69]
NOX-A12	A mirror-image oligonucleotide inhibitor of CXCL12	Single-dose NOX-A12 exhibits comparable mobilization effects to that of AMD3100; NOX-A12 synergizes with G-CSF to enhance mobilization.	Vater et al. [80]
Me6^ab^	An alkaloid analog that can disrupt the SDF-1α/CXCR4 interaction	Single-dose Me6 was more effective in mobilization than AMD3100 or G-CSF alone; Me6 synergized with G-CSF in mobilization with higher efficiency than G-CSF + AMD3100.	Zhang et al. [85]
LGB321	A PIM1 kinase inhibitor that can regulate CXCR4 expression	LGB321 enhanced AMD3100-induced mobilization.	Müller et al. [109]
**3. Agents targeting VLA-4 (α4β1)/VCAM-1 axis**			
**3.1 VLA-4 antagonist**			
BIO5192	A VLA-4 antagonist	Single-dose BIO5192 alone induced mobilization; BIO5192 enhanced mobilization response when combined with G-CSF, AMD3100, or tGro-β.	Ramirez et al. [64], Cao et al. [90], Karpova et al. [107]
Thioridazine	An allosteric antagonist of VLA-4	The mobilizing ability of thioridazine was comparable to AMD3100.	Chigaev et al. [70]
BOP	A dual α9β1/α4β1 integrin antagonist	Single-dose BOP alone induced rapid mobilization comparable to that induced by AMD3100; BOP synergizes with G-CSF and AMD3100 in mobilization.	Cao et al. [90]
CWHM-823 and -842	VLA-4 antagonists	Single-dose CWHM-823 or -842 induced mobilization; the combination of CWHM-823 or -842 with tGro-β enhanced mobilization.	Karpova et al. [107]
Firategrast	A VLA-4 antagonist	Single-dose firategrast induced mobilization; the combination of firategrast with tGro-β enhanced mobilization.	Karpova et al. [107]
**3.2 Other agents targeting VLA-4/VCAM-1 axis**			
Anti-VCAM-1 Ab	Antibody of VCAM-1	Anti-VCAM-1 Ab alone can induce mobilization; the combination of Anti-VCAM-1 Ab with G-CSF increased mobilization.	Kikuta et al. [32], Saez et al. [84]
Bortezomib	A proteasome inhibitor that can inhibit transcription and expression of VCAM-1	Single-dose bortezomib induced significant mobilization; Bortezomib enhanced the mobilization effect of G-CSF and AMD-3100.	Ghobadi et al. [82]
Ixazomib	A novel proteasome inhibitor that is speculated to modulate VLA4/VCAM1 axis as bortezomib	Single-dose ixazomib can induce mobilization; ixazomib synergizes with G-CSF but not AMD3100 to enhance mobilization.	Ghobadi et al. [100]
**4. Heparan sulfate**			
Fucoidan	A sulfated polysaccharide that can competitively displace SDF-1 from heparan sulfate proteoglycan anchors.	Fucoidan alone induced rapid HSPC mobilization. Fucoidan works synergistically with G-CSF in mobilization.	Frenette et al. [31], Sweeney et al. [34], Sweeney et al. [39], Albanese et al. [60]
EP80031	A heparan sulfate mimetic that can compete with endogenous heparan sulfate.	Single-dose EP80031 alone induced rapid mobilization with efficacy comparable to G-CSF and AMD 3100; EP80031 can act synergistically with G-CSF and AMD 3100 to mobilize HSPCs.	di Giacomo et al. [72]
Heparin	A pharmacological competitive inhibitor of heparan sulfate	Heparin alone only induced modest mobilization; heparin plus G-CSF increased the mobilization of long-term reconstituting and efficient self-renewing cells.	Saez et al. [84]
**5. Agents targeting purinergic signaling**			
AMP, ATP	Extracellular nucleotides	Combination of AMP with DP induced significant mobilizing effects; ATP enhanced G-CSF- and AMD 3100-induced mobilization	Hofer et al. [41], Adamiak et al. [99]
DP	Dipyridamole, a drug inhibiting the cellular uptake of adenosine	DP + AMP induced significant mobilizing effects.	Hofer et al. [41]
ARL67156, AMPCP	Inhibitor of cell surface ectonucleotidase CD39 or CD73	Both ARL67156 and AMPCP can enhance G-CSF- and AMD 3100-induced mobilization	Adamiak et al. [104]
**6. Agents inhibiting Cdc42 activity**			
Erlotinib	An EGFR inhibitor that can reduce Cdc42 activity	Erlotinib alone did not induce mobilization; erlotinib enhanced G-CSF-mediated mobilization.	Ryan et al. [67]
ML141	A Cdc42 inhibitor	ML141 alone only induced modest mobilization but played a synergistic effect in G-CSF-mediated mobilization.	Chen et al. [81]
CASIN	A Cdc42 activity-specific inhibitor	Single-dose CASIN alone can induce mobilization; CASIN enhanced G-CSF- and AMD 3100-induced mobilization.	Liu et al. [108]
**7. Agents targeting sympathetic nervous system signaling**			
Desipramine, reboxetine	Norepinephrine reuptake inhibitors	Desipramine alone did not induce mobilization; desipramine and reboxetine enhanced G-CSF-induced mobilization but did not affect AMD3100-induced mobilization.	Lucas et al. [74]
Adrenaline	Catecholaminergic neurotransmitter	Adrenaline alone did not induce mobilization; adrenaline enhanced the mobilization efficiency of G-CSF.	Chen et al. [75]
NE	Norepinephrine, catecholaminergic neurotransmitter	NE alone can induce mobilization; NE enhanced AMD3100-induced mobilization.	Dar et al. [71]
**8. Agents targeting S1P signaling**			
SEW2871	A S1PR1 agonist	SEW2871 alone did not induce mobilization; administration of SEW2871 enhanced AMD3100- but not G-CSF-mediated mobilization.	Juarez et al. [73], Ogle et al. [96]
VPC01091	A selective S1PR3 antagonist	VPC01091 alone can induce mobilization; VPC01091 enhanced AMD3100-mediated mobilization.	Ogle et al. [96]
THI	An inhibitor of sphingosine phosphate lyase	THI enhanced mobilization induced by G-CSF and AMD3100.	Adamiak et al. [93]
SLM6031434	An inhibitor of sphingosine kinase type 2	SLM6031434 enhanced mobilization induced by G-CSF and AMD3100.	Adamiak et al. [93]
Anti-CD69 Ab	An antibody of CD69 that can increase S1PR1 expression	Anti-CD69 Ab induced mobilization of the same magnitude as AMD3100 but did not synergize with AMD3100.	Notario et al. [102]
**9. Other agents**			
CY, paclitaxel and docetaxel	Chemotherapeutic agents	Priming with cyclophosphamide, paclitaxel, or docetaxel induced mobilization and enhanced G-CSF-induced mobilization.	Neben et al. [19], Verma et al. [29], Ojeifo et al. [43]
PGG-glucan	A polysaccharide	Single-dose PGG-glucan alone can induce mobilization; PGG-glucan enhanced G-CSF-mediated mobilization.	Patchen et al. [26], Cramer et al. [28]
ProGP	Progenipoietin-1, an agonist of both the G-CSF and FLT-3 receptors	ProGP-mobilized cells exhibited greater spleen colony-forming activity and competitive repopulating activity than that of G-CSF.	Fleming et al. [35]
Defibrotide	A polydeoxyribonucleotide	Defibrotide alone had no mobilizing activity, addition of defibrotide significantly enhanced G-CSF-induced mobilization.	Carlo-Stella et al. [38]
α-LFA-1, α-Mac-1	Antibody of β2 integrin LFA-1 or Mac-1	The antibodies themselves had no mobilizing capacity; α-LFA-1 and α-Mac-1 increased G-CSF-induced mobilization.	Velders et al. [40]
Anti-CD49d Ab	Antibody of CD49d	5-day anti-CD49d Ab alone can induce mobilization; anti-CD49d Ab enhanced G-CSF-induced mobilization.	Liu et al. [45]
s-kit	A soluble form of c-kit receptor	s-kit alone can induce mobilization; s-kit increased G-CSF-induced mobilization.	Nakamura et al. [46]
uPAR_84-95_	A derived chemotactic peptide of the cleaved forms of soluble uPAR	2-day uPAR_84-95_ exhibited mobilization potency similar to that of 5-day G-CSF; uPAR_84-95_ did not act synergistically or additively with G-CSF.	Selleri et al. [50]
VTP195183	A RARα specific agonist	VTP195183 alone did not induce mobilization; VTP195183 synergizes with G-CSF to enhance mobilization.	Herbert et al. [55]
Anti-Notch2 Ab	Antibody of Notch2	Single-dose anti-Notch2 Ab enhanced G-CSF- and AMD3100-induced mobilization.	Wang et al. [98]
PTH	Parathyroid hormone	6-day PTH alone can induce mobilization; a combination of PTH and G-CSF showed slight additional effects.	Brunner et al. [57]
Tenecteplase, microplasmin	Thrombolytic agents	Tenecteplase and microplasmin enhanced G-CSF-induced mobilization.	Tjwa et al. [59]
OTR_4120_, OTR_4131_	Glycosaminoglycan mimetics	Single-dose OTR_4120_ or OTR_4131_ can induce mobilization as effectively as G-CSF and AMD3100; they synergize with G-CSF or AMD3100 in mobilization.	Albanese et al. [60]
Im-HD	Immobilized hyaluronidase	The native hyaluronidase and Im-HD alone did not induce significant mobilization; Im-HD enhanced G-CSF-induced mobilization.	Dygai et al. [76]
Meloxicam, indomethacin	NSAIDs	Meloxicam or indomethacin alone can induce mobilization; meloxicam and indomethacin enhanced G-CSF- and AMD 3100-induced mobilization.	Hoggatt et al. [101]
AH23848 and L-161,982	EP4 receptor antagonists	Co-administration of AH23848 or L-161,982 with G-CSF significantly enhanced mobilization.	Hoggatt et al. [101]
UDP-G	Uridine diphosphate-glucose	UDP-G showed comparable mobilizing ability to G-CSF; the combination of UDP-G and G-CSF enhanced mobilization.	Kook et al. [79]
FG-4497	A HIF-1α PHD inhibitor	FG-4497 alone did not induce mobilization; FG-4497 synergizes with G-CSF and AMD 3100 to enhance mobilization.	Forristal et al. [86], Nowlan et al. [95], Bisht et al. [106]
CasNa	Sodium caseinate	Four-dose CasNa induced significant mobilization.	Santiago-Osorio et al. [87]
SnPP	Tin protoporphyrin IX, an inhibitor of HO-1	SnPP significantly increased G-CSF- and AMD 3100-induced HSPC mobilization.	Wysoczynski et al. [88]
HS6101	A small molecule lipopeptide	Single-dose HS6101 alone can induce mobilization.	Xing et al. [89]
Dexamethasone	Glucocorticoid	Dexamethasone enhanced AMD3100-induced mobilization.	Yan et al. [92]
Viagra	Sildenafil citrate	Single-dose Viagra did not induce mobilization, but significantly improved AMD3100-induced mobilization.	Smith-Berdan et al. [110]
CoPP	Cobalt protoporphyrin IX	5-day CoPP induced mobilization more efficiently than G-CSF.	Szade et al. [111]

^a^Agents with superior mobilization potentials compared with G-CSF

^b^Agents with superior mobilization potentials compared with AMD3100

*Abbreviations*: *AMP* adenosine monophosphate, *ATP* adenosine triphosphate, *Cdc42* Cell division control protein 42, *CSF1* Colony-stimulating factor 1, *CXCR-4* C-X-C chemokine receptor type 4, *CY* cyclophosphamide, *EGFR* Epidermal growth factor receptor, *EP4* E-proteinoid 4, *G-CSF* Granulocyte colony-stimulating factor, *HIF-1α* Hypoxia-inducible transcription factor 1α, *HO-1* Heme oxygenase 1, *HSPCs* hematopoietic stem and progenitor cells, *LFA-1* Leukocyte function antigen-1, *Mac-1* macrophage antigen-1, *NSAIDs* nonsteroidal anti-inflammatory drugs, *PHD* Prolyl hydroxylase domain enzyme, *PIM1* Proviral integration site for Moloney murine leukemia virus, *RARα* Retinoic acid receptor alpha, *SDF-1* Stromal cell-derived factor-1, *S1PR1* Sphingosine-1-phosphate receptor 1, *S1PR3* Sphingosine-1-phosphate receptor 3, *THI* Tetrahydroxybutylimidazole, *uPAR* urokinase receptor, *VCAM-1* Vascular cell adhesion molecule-1, *VLA-4* Very late antigen-4

After excluding studies using non-C57BL/6 mice model, studies including mice older than 12 weeks, studies that did not reported data about the number of total CFCs or LSK cell per milliliter PB, studies with significant heterogeneity in G-CSF dosage, and studies that were unrelated to the main network, 21 eligible studies were included in meta-analysis [51, 56, 72, 74, 77, 78, 81–86, 91, 94, 96, 104–106, 108, 109, 112]. All of the 21 included studies are controlled studies, and the most widely used controls are phosphate-buffered saline (PBS), saline, and G-CSF. There are 40 mobilization agents and 57 regimens investigated. The characteristics of these 21 studies are summarized in Table 2. The results of methodological quality evaluation are listed in Supplementary Table 2. Risk of bias regarding random allocation and blinding in all included studies are unclear since the lack of relevant information. The baseline characteristics including mice strain and gender are unified among groups in 10 studies, the other 11 studies did not report the animal gender and age. There are 2 studies that only reported representative data for mobilization outcomes; the other studies are all free from bias caused by incomplete outcome data, selective outcome reporting, and other reasons.

**Table 2:** Characteristics of the 21 studies included in meta-analysis

Study	Mice characteristics	Experimental arm	Dose
Abraham et al. [51]	C57BL/6, Female, 7–8 weeks	Long-term SD G-CSF; AMD3100; T-140; long-term SD G-CSF + T-140; long-term SD G-CSF + AMD3100	G-CSF, 2.5 μg/mouse s.c. twice daily for 4 days; AMD3100 5 mg/kg s.c. 2 h before harvest; T-140 5 mg/kg s.c. 2 h before harvest
Kubonishi et al. [56]	C57BL/6, male and female, 7–12 weeks	SCA; long-term SD G-CSF; long-term SD G-CSF + SCA	G-CSF, 125 μg/kg s.c. twice daily for 4 days; SCA, 100 mg/kg i.v. 30 min before harvest
di Giacomo et al. [72]	C57BL/6	EP80031; long-term SD G-CSF + EP80031; AMD3100 + EP80031; long-term SD G-CSF + AMD3100; long-term SD G-CSF + AMD3100 + EP80031	EP80031, 15 mg/kg i.v. 1 h before harvest; G-CSF, 2.5 μg/mouse s.c. twice daily for 4 days; AMD1300, 5 mg/kg s.c. 1 h before harvest
Lucas et al. [74]	C57BL/6, male, 8 weeks	Long-term SD G-CSF; long-term SD G-CSF + desipramine; AMD3100; AMD3100 + desipramine; long-term SD G-CSF + reboxetine	G-CSF, 125 μg/kg s.c. twice daily for 4 days; AMD 3100, 5 mg/kg s.c. 1 h before collection; desipramine, 10 mg/kg/day i.p. for 8 days; reboxetine, 5 mg/kg/day i.p. for 8 days
Hoggatt et al. [77]	C57BL/6	Meloxicam; indomethacin; long-term LD G-CSF; AMD3100; long-term LD G-CSF + indomethacin; long-term LD G-CSF + meloxicam; AMD3100 + meloxicam	G-CSF, 50 μg/kg s.c. twice daily for 4 days; AMD 3100, 5 mg/kg i.p. 1 h before collection; meloxicam, 0.5–12 mg/kg s.c. for 4 days; indomethacin, 0.5–2.5 mg/kg s.c. twice daily for 4 days; AH23848, 10 μg per mouse i.p. for 4 days; L-161,982, 10 μg per mouse i.p. for 4 days
Karpova et al. [78]	C57BL/6	POL5551; HD POL5551; AMD3100	POL5551, 5 or 100 (HD) mg/kg i.p. 2 or 4 h before harvest; AMD3100, 5 mg/kg i.p. 1 h before harvest.
Chen et al. [81]	C57BL/6	Long-term SD G-CSF; ML141; long-term SD G-CSF + ML141	G-CSF, 200 μg/kg/day s.c. for 5 days; ML141, 10 μg/kg/day i.p. for 5 days
Ghobadi et al. [82]	C57BL/6	Bortezomib; long-term SD G-CSF; AMD3100; long-term SD G-CSF + Bortezomib; AMD3100 + Bortezomib	Bortezomib, a single dose of 0.8mg/kg i.v.; G-CSF, 250 μg/kg/day s.c. for 4 days; AMD3100, 5 mg/kg s.c.
He et al. [83]	C57BL/6, 8–10 weeks	10d-FLT-3L; short-term SD G-CSF + AMD3100; 10d-FLT-3L + AMD3100	FLT-3L, 350 μg/kg/day i.p. for 10 days; G-CSF, 150 μg/kg/day i.p. for 5 days; AMD3100, 5 mg/kg i.p. 1 h before harvest
Saez et al. [84]	C57BL/6, male, 6–12 weeks	Long-term SD G-CSF; long-term SD G-CSF + heparin; G-CSF + Anti-VCAM-1 Ab; AMD310; heparin; AMD3100 + heparin	G-CSF, 125 μg/kg s.c. twice for 4 days; heparin, 100 U i.p. 1 h before harvest; Anti-VCAM-1 Ab, 2 mg/kg/day i.v. for 3 days; AMD3100, 5 mg/kg s.c. 1 h before harvest
Zhang et al. [85]	C57BL/6	Me6; AMD3100; long-term SD G-CSF; long-term SD G-CSF + AMD3100; long-term SD G-CSF + Me6	Me6, 5 mg/kg s.c. 12 h before harvest; AMD3100, 5mg/kg s.c. 1 h before harvest; G-CSF, 2.5 μg per mouse s.c. twice daily for 4 days
Forristal et al. [86]	C57BL/6, male, 9–12 weeks	Long-term SD G-CSF; long-term SD G-CSF + AMD3100; long-term SD G-CSF + FG-4497; long-term SD G-CSF + AMD3100 + FG-4497	G-CSF, 125 μg/kg s.c. twice daily for 4 days; AMD3100, 5 mg/kg s.c. 1 h before harvest; FG-4497, 20 mg/kg/day i.p. for 3 days
Lu et al. [91]	C57BL/6, male, 6–8 weeks	LECT2; AMD3100; AMD3100 + LECT2	LECT2, 300 μg/kg/day s.c. for 5 days; AMD 3100, 5 mg/kg s.c. 1 h before collection
Karpova et al. [94]	C57BL/6	HD POL5551; 14d-HD POL5551; 14d-HD AMD3100; 14d-ALT1188; 14d-HD POL5551 + HD AMD3100; 14d-HD POL5551 + CWHM-823	POL5551, 100mg/kg i.p. as a single dose or as continuous infusion for 2 weeks via subcutaneously implanted pumps; ALT1188, 33 mg/kg i.p. as a single injection or as continuous infusion for 2 weeks; AMD3100, 20 mg/kg i.p. as a single injection or as continuous infusion for 2 weeks; CWHM-823, 3 mg/kg i.p.
Ogle et al. [96]	C57BL/6, male, 8–12 weeks	VPC01091; AMD3100; AMD3100 + VPC01091	AMD3100 5mg/kg i.p. 1.5 h before harvest; VPC01091 5mg/kg i.p. 1.5 h before harvest
Adamiak et al. [104]	C57BL/6, 4–6 weeks	Short-term LD G-CSF; AMD3100; short-term LD G-CSF + ARL67156; AMD3100 + ARL67156; short-term LD G-CSF + AMPCP; AMD3100 + AMPCP	G-CSF, 100 μg/kg/day s.c. for 3 days; AMD 3100, 5 mg/kg i.p. 1 h before collection; ARL67156, 2 mg/kg i.p.; AMPCP, 4 mg/kg i.p.
Alt et al. [105]	C57BL/6, Male, 6–10 weeks	Short-term SD G-CSF; AMD3100; short-term SD G-CSF + AMD3100; IL-33; short-term SD G-CSF + IL-33; AMD 3100 + IL-33; short-term SD G-CSF + AMD3100 + IL-33	G-CSF, 200 μg/kg/day s.c. for 3 days; AMD 3100, 5 mg/kg i.p. 1 h before collection; IL-33, 0.04 mg/kg/day i.p. for 3 days
Bisht et al. [106]	C57BL/6, male, 8–9 weeks	Short-term SD G-CSF; short-term SD G-CSF + FG-4497	G-CSF, 125 μg/kg s.c. twice daily for 2 days; FG-4497, 20 mg/kg/day i.p. for 3 days;
Liu et al. [108]	C57BL/6	CASIN; AMD3100; CASIN + AMD3100	CASIN, 1.2mg/kg i.v. 2 h before harvest; AMD3100, 5mg/kg i.p. 2 h before harvest.
Müller et al. [109]	C57BL/6	AMD3100; AMD3100 + LGB321	AMD3100, 5 mg/kg s.c.; LGB321, 100 mg/kg s.c.
Fang et al. [112]	C57BL/6	HF51116; AMD 3100; long-term SD G-CSF; G-CSF + HF51116; long-term SD G-CSF + AMD 3100	G-CSF, 100 μg/kg every 12h s.c. for 4 days; AMD 3100, 5 mg/kg s.c.; HF51116, 5 mg/kg s.c.

*Abbreviations*: *ALT1188* a small molecule CXCR4 antagonist; *AMPCP* an inhibitor of cell surface ectonucleotidase CD73; *ARL67156* an inhibitor of cell surface ectonucleotidase CD39; *CFCs* colony-forming cells; *CASIN* cell division control protein 42 (Cdc42) activity-specific inhibitor; *CWHM-823* small molecule very late antigen 4 (VLA4) antagonist; *EP80031* synthetic octo-saccharides, a heparan sulfate mimetic; *FG-4497* hypoxia-inducible transcription factor prolyl hydroxylase domain enzymes inhibitor; *FLT-3L* fms-like tyrosine kinase-3 ligand; *G-CSF* granulocyte colony-stimulating factor; *HD* high dose; *HF51116* a new CXCR4 antagonist; *IL-33* interleukin 33; *LD* low dose; *LGB321* Proviral integration site for Moloney murine leukemia virus (PIM) kinase inhibitor; *LSK cells* Lin^−^ Sca1^+^ Kit^+^ cells; *Me6* Me6TREN, Tris[2-(dimethylamino)ethyl]amine); *ML141* cell division control protein 42 (Cdc42) inhibitor; *PEGLip-G-CSF* pegylated liposome formulated granulocyte colony-stimulating factor; *POL5551* a peptidic CXCR4 antagonist; *SCA* sulfated colominic acid; *SD* standard dose; *T-140* 4F-benzoyl-TN14003, a highly selective CXCR4 antagonist; *VPC01091* a selective sphingosine-1-phosphate receptor 3 antagonist

### Total CFCs

**Fig. 2.**
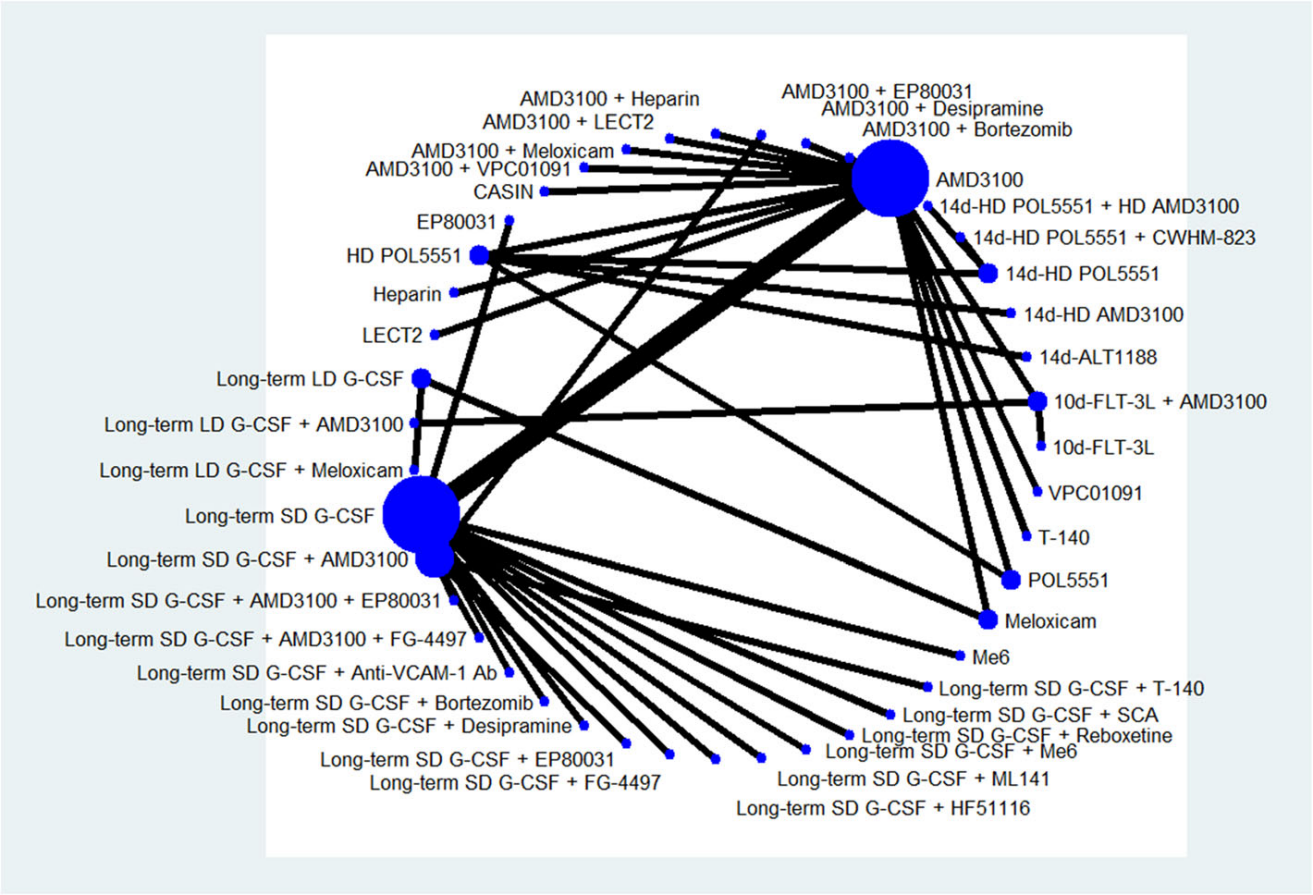
Network graph for total CFCs. The network graph of all comparisons in the 21 studies that have data about total colony-forming cells (CFCs) per milliliter of peripheral blood (/ml PB). Each node represents a mobilization regimen, while each line represents a direct comparison between regimens, with the thickness reflecting the number of available direct comparisons. All included regimens are described in the supplementary materials

**Fig. 3:**
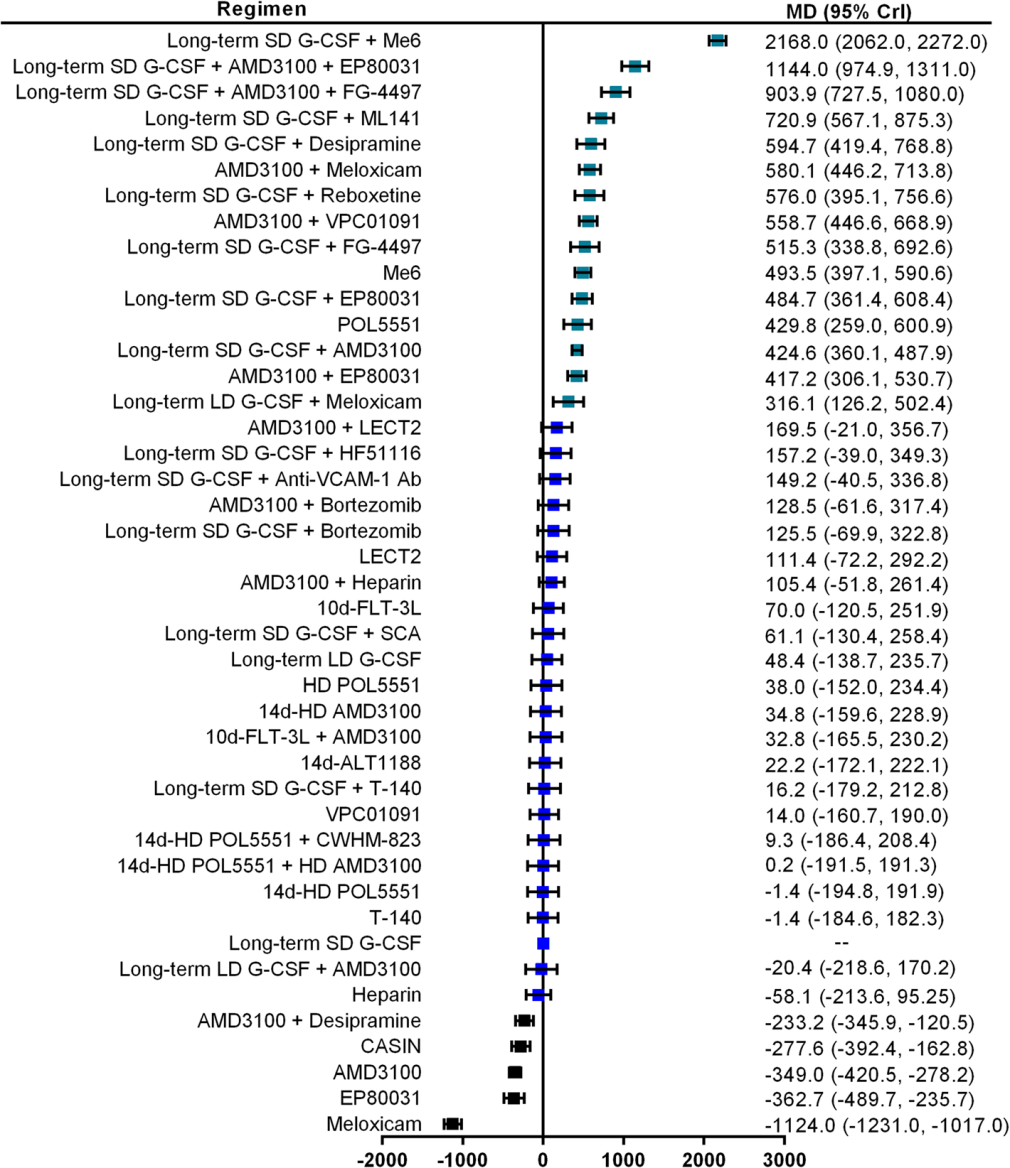
Forest plots for total CFCs. Forest plot of the Bayesian network meta-analysis results about the number of harvested total colony-forming cells (CFCs) per milliliter of peripheral blood (/ml PB). Estimate of treatment effect for each mobilization regimen was reported as mean differences (MD) with the associated 95% credibility interval (95% CrI). Granulocyte colony-stimulating factor monotherapy (G-CSF) is a common comparator. All included regimens are described in the supplementary materials

The number of total CFCs (also known as colony-forming units, CFUs) per milliliter of PB was reported as primary outcome in 17 studies and involved 43 mobilization regimens. The network graph of all comparisons in these 17 studies is shown in Fig. 2. The results of Bayesian network meta-analysis indicate that compared with long-term SD G-CSF alone, 14 mobilization regimens significantly increased the number of total CFCs/ml PB, including long-term SD G-CSF + Me6 (MD 2168.0, 95% CrI 2062.0–2272.0), long-term SD G-CSF + AMD3100 + EP80031 (MD 1144.0, 95% CrI 974.9–1311.0), long-term SD G-CSF + AMD3100 + FG-4497 (MD 903.9, 95% CrI 727.5, 1080.0), long-term SD G-CSF + ML141 (MD 720.9, 95% CrI 567.1–875.3), long-term SD G-CSF + desipramine (MD 594.7, 95% CrI 419.4–768.8), AMD3100 + meloxicam (MD 580.1, 95% CrI 446.2–713.8), long-term SD G-CSF + reboxetine (MD 576.0, 95% CrI 395.1–756.6), AMD3100 + VPC01091 (MD 558.7, 95% CrI 446.6–668.9), long-term SD G-CSF + FG-4497 (MD 515.3, 95% CrI 338.8–692.6), Me6 (MD 493.5, 95% CrI 397.1–590.6), long-term SD G-CSF + EP80031 (MD 484.7, 95% CrI 361.4–608.4), POL5551 (MD 429.8, 95% CrI 259.0–600.9), long-term SD G-CSF + AMD3100 (MD 424.6, 95% CrI 360.1–487.9), AMD1300 + EP80031 (MD 417.2, 95% CrI 306.1–530.7), and long-term LD G-CSF + meloxicam (MD 316.1, 95% CrI 126.2, 502.4) (Fig. 3). Long-term SD G-CSF + Me6 ranked first among these regimens in regard to the ability to mobilize CFCs. AMD1300 + desipramine, Cdc42 activity-specific inhibitor (CASIN) alone, AMD3100 alone, EP80031 alone, and meloxicam alone are inferior to long-term SD G-CSF. No significant differences are identified between the other regimens and long-term SD G-CSF.

### LSK cells

**Fig. 4:**
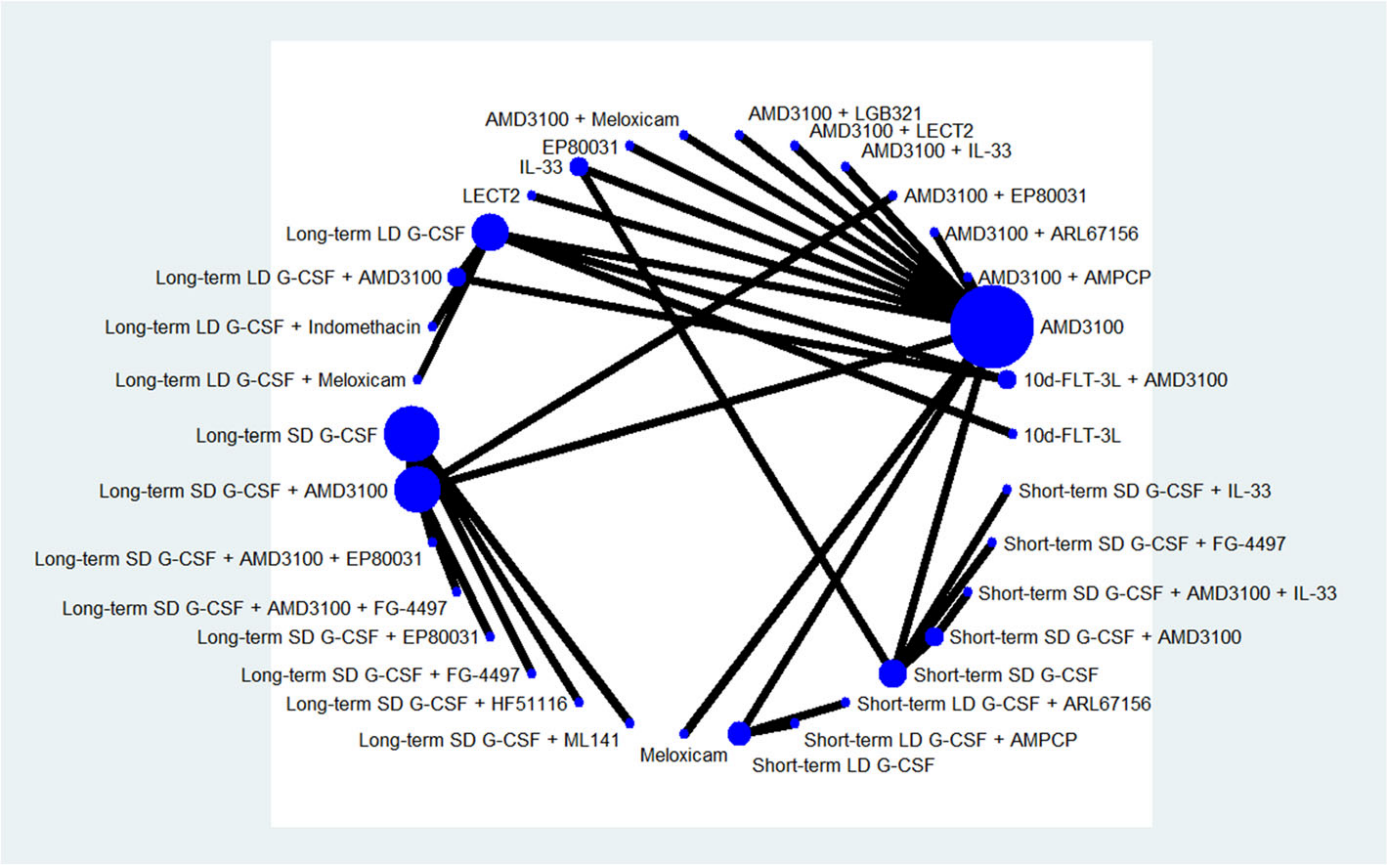
Network graph for LSK cells. The network graph of all comparisons in the 10 studies that have data about Lin^−^ Sca1^+^ Kit^+^ (LSK) cells per milliliter of peripheral blood (/ml PB). Each node represents a mobilization regimen, while each line represents a direct comparison between regimens, with the thickness reflecting the number of available direct comparisons. All included regimens are described in the supplementary materials.

**Fig. 5:**
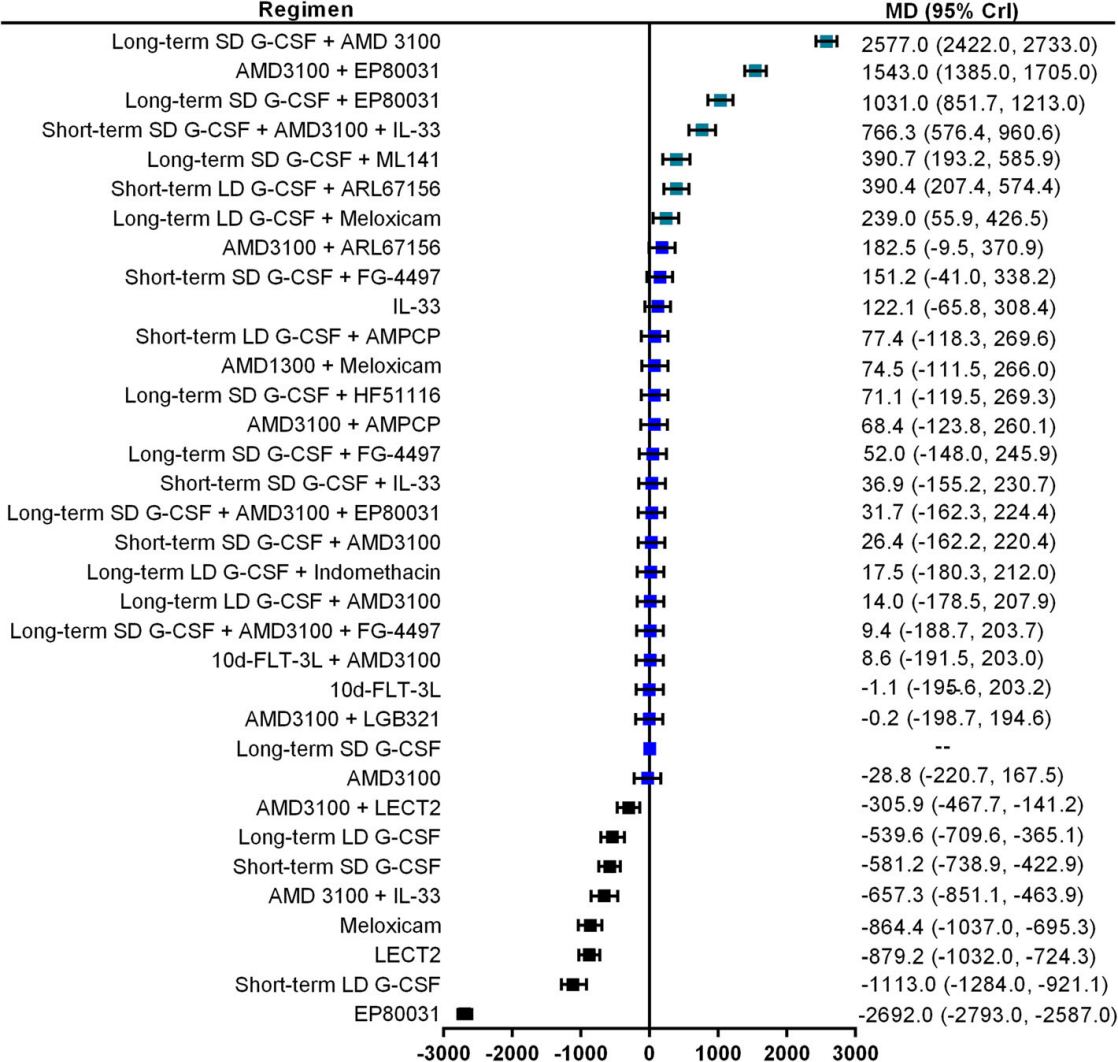
Forest plots for LSK cells. Forest plot of the Bayesian network meta-analysis results about the number of harvested Lin^−^ Sca1^+^ Kit^+^ (LSK) cells per milliliter of peripheral blood (/ml PB). Estimate of treatment effect for each mobilization regimen was reported as mean differences (MD) with the associated 95% credibility interval (95% CrI). Granulocyte colony-stimulating factor monotherapy (G-CSF) is a common comparator. All included regimens are described in the supplementary materials

The number of LSK cells/ml PB was reported as primary outcome in 11 studies, which have evaluated the efficacy of 34 mobilization regimens with mice models. The network graph of all comparisons in these 11 studies is shown in Fig. 4. The results of Bayesian network meta-analysis indicate that in comparison with long-term SD G-CSF alone, 7 mobilization regimens significantly increased the number of LSK cells collected from peripheral blood, including long-term SD G-CSF + AMD3100 (MD 2577.0, 95% CrI 2422.0–2733.0), AMD3100 + EP80031 (MD 1543.0, 95% CrI 1385.0–1705.0), long-term SD G-CSF + EP80031 (MD 1031.0, 95% CrI 851.7–1213.0), short-term SD G-CSF + AMD3100 + IL-33 (MD 766.3, 95% CrI 576.4–960.6), long-term SD G-CSF + ML141(MD 390.7, 95% CrI 193.2–585.9), short-term LD G-CSF + ARL67156 (MD 390.4, 95% CrI 207.4–574.4), and long-term LD G-CSF + meloxicam (MD 239.0, 95% CrI 55.9–426.5). The MD and 95% CrI of all included regimens are presented in forest plot in the order of median rank (Fig. 5). Long-term SD G-CSF + AMD3100 ranked first among these regimens considering this parameter since it is associated with most favorable MD and ranked first in all simulations. AMD3100 + LECT2, long-term LD G-CSF, short-term SD G-CSF, AMD3100 + IL-33, meloxicam alone, LECT2 alone, short-term LD G-CSF, and EP80031 alone are inferior to G-CSF in regard to the ability of mobilizing LSK cells into blood. No significant differences are identified between the other regimens and long-term SD G-CSF.

### Long-term repopulating ability

Although the number of CFCs and LSK cells are the most commonly used outcomes to evaluate HSPC mobilization efficiency, enriched cell subsets such as CFCs and LSK cells do not measure long-term reconstituting HSCs, and additional markers such as fms-like tyrosine kinase-3 (Flt3) and signaling lymphocyte activation molecule (SLAM) CD150 were used to identify LSK subsets and assess the mobilization of self-renewing HSCs and long-term HSCs (LT-HSCs) [114]. The mobilization of different LSK subsets were examined in 12 studies, and the results are summarized in Supplementary Table 3. In brief, combination of desipramine, meloxicam, hypoxia-inducible transcription factor 1α (HIF-1α) prolyl hydroxylase domain enzyme (PHD) inhibitors (FG-4497, PHI-1, or PHI-2), the dual α9β1/α4β1 integrin antagonist BOP, Viagra, new CXCR4 antagonist HF51116, and colony-stimulating factor 1 Fc fusion protein (CSF1-Fc) significantly increased the mobilization of LSKF cells (Lin^−^Sca-1^+^c-kit^+^Flt3^−^ cells), SLAM LSK cells (Lin^−^Sca-1^+^c-kit^+^ CD48^−^CD150^+^ cells), or LT-HSCs compared with G-CSF alone. The truncated form of chemokine GROβ (tGROβ) plus AMD3100, Cobalt protoporphyrin IX (CoPP), mobilized higher levels of SLAM LSK cells than G-CSF.

To further assess the mobilization of long-term repopulating HSCs, in vivo transplantation experiments were performed in 49 studies. The characteristics and results of these 49 studies are reviewed in Supplementary Table 4. In summary, lethally irradiated recipient mice received mobilized PB cells from donor mice with or without competitive cells, and the long-term repopulating ability are assessed by the survival of recipients and the long-term reconstitution donor-derived cells at different time point (usually in at months after transplantation). Furthermore, serial transplantation analysis was performed via transplanting BM cells from primary recipients to secondary or tertiary recipients to assess the long-term repopulating and self-renewing capacity of mobilized cells in 20 studies. Results indicate that the combination of new mobilization agents (including FLT-3L, MIP-1α, IL-8, PEG-rHuMGDF, SB-251353, s-kit, AMD3100, T-140, tGROβ, VTP195183, SCA, erlotinib, EP80031, meloxicam, UDP-G, Anti-VCAM-1 Ab, heparin, Me6, HF51116, and CSF1-Fc) significantly increased the mobilization of long-term repopulating HSCs compared with G-CSF alone. In addition, the combination of BOP, BIO5192, SEW2871, VPC01091, LGB321, or Viagra with AMD3100 enhanced the mobilization of long-term repopulating HSCs compared with AMD3100 alone. Moreover, cells mobilized by LECT2, POL5551, UDP-G, or CoPP alone showed superior long-term repopulating capacity than those mobilized by G-CSF, whereas cells mobilized by Me6, CasNa, or CASIN alone showed superior long-term repopulating capacity than those mobilized by AMD3100.

## Discussion

This work reviewed the efficacy of 94 new HSC mobilization agents from 95 preclinical studies. In addition, we included 21 studies using the poor mobilizer model-C57BL/6 mice for network meta-analysis and compared the efficacy of 57 mobilization regimens. We identified several promising regimens with great HSC mobilization efficacy, including long-term SD G-CSF + Me6, long-term SD G-CSF + AMD3100 + EP80031, long-term SD G-CSF + AMD3100 + FG-4497, long-term SD G-CSF + ML141, long-term SD G-CSF + desipramine, AMD3100 + meloxicam, long-term SD G-CSF + reboxetine, AMD3100 + VPC01091, long-term SD G-CSF + FG-4497, Me6, POL5551, long-term SD G-CSF + AMD3100, long-term LD G-CSF + meloxicam, AMD3100 + EP80031, long-term SD G-CSF + EP80031, short-term SD G-CSF + AMD3100 + IL-33, and short-term LD G-CSF + ARL67156.

To our best of knowledge, this study is the first network meta-analysis that compared the efficacy of different HSC mobilization regimens with data from preclinical studies. We provide a comprehensive summary of new mobilization agents that have been investigated in mice models. The efficacy of these agents alone or in combination with other agents was indirectly compared via network meta-analysis. Moreover, we ranked all of the investigated regimens based on their ability to mobilize HSCs into blood stream. We identified several promising agents and regimens that have the most potent mobilizing capacity. The majority of mobilization regimens that show great improvements over G-CSF are combined regimens containing both G-CSF and new mobilization agents. Although these regimens would be unlikely to reduce severe adverse events, they provide a perspective that the incorporation of new agents could reduce the incidences of G-CSF-related adverse events through reducing the doses of G-CSF that required to mobilization sufficient HSCs since they can synergistically enhance the G-CSF-mediated mobilization. In addition, we identified several agents showed superior mobilization potential than G-CSF even when used alone, such as Me6 and POL5551. It is worth further investigation that whether these agents could reduce mobilization-related toxicity compared with G-CSF.

Among the new agents, EP80031, Me6, FG-4497, and ML141 significantly improved the efficiency of G-CSF-induced HSC mobilization. EP80031 is a synthetic octosaccharide mimicking the structure of heparan sulfate. A single dose of EP80031 (15mg/kg, intravenously injection) could lead to rapid and prominent mobilization of hematopoietic stem and progenitor cells (HSPCs), and the combination of EP80031 with G-CSF and AMD3100 resulted in 3-fold increase in the number of LSK cells and total CFCs [72]. In addition, HSCs mobilized with the regimen G-CSF + AMD3100 + EP80031 are associated with enhanced hematopoietic reconstitution [72]. Me6 is a small molecule that was screened from a group of chemicals by Zhang et al*.* and has been proved to have robust ability of mobilizing HSPCs [85]. The combination of Me6 and G-CSF (G-CSF + Me6) resulted in remarkable increase in the number of total CFUs, moreover, it is suggested that Me6-mobilized HSCs are associated with greater long-term repopulating capacity and more efficient engraftment [85]. FG-4497 is a prolyl hydroxylase inhibitor that could enhance HSC mobilization through stabilizing the hypoxia-inducible transcription factor-1α (HIF-1α) protein [106]. The addition of FG-4497 significantly increased the mobilization of HSPCs induced by G-CSF [86, 106]. In addition, FG-4497 exerts protective effects in ischemia-induced kidney injury and high-dose irradiation-induced BM failure [115]. ML141 is an inhibitor of cell division control protein 42 (Cdc42). The mobilization effect of ML141 is modest, but ML141 could synergistically enhance G-CSF-mediated mobilization of LSK cells and CFCs in mice model [81]. Taking our results of meta-analysis together into consideration, G-CSF + AMD3100 + EP80031, G-CSF + Me6, G-CSF + FG-4497, and G-CSF + ML141 are new promising mobilization regimens that could significantly increase the quantity of HSCs in PB without interfering their functions. However, the safety profiles of these new agents remain unclear. Further studies are required to determine the efficacy and safety of these potential regimens in human before applied in clinical practice.

In addition, we established the favorable efficacy of G-CSF and AMD3100 in HSC mobilization, which has been verified by clinical trials. AMD3100, also known as plerixafor, is an antagonist of the chemokine receptor CXCR4 that could rapidly induce the mobilization of stem cells through antagonizing the interaction of CXCR4 and stromal cell-derived factor-1α (SDF-1α) [116]. Multiple studies have demonstrated that AMD3100 alone mobilized lower numbers of HSCs compared with G-CSF, but the addition of AMD3100 dramatically increased the G-CSF-induced mobilization of HSCs both in mice models and non-human primates’ model [117, 118]. Our results from network meta-analyses indicated that G-CSF in combination of AMD3100 not only significantly increased the number of LSK cells, but also increased total CFCs. Despite we only pooled data from murine models, which are different from human in regard to physiological conditions, our conclusions are consistent with that obtained from clinical studies in human beings. A group of randomized controlled trials (RCTs) have demonstrated that G-CSF in combination of AMD3100 led to higher rates of successful mobilization and increased the total collection of HSCs without increasing the risk of severe adverse events in patients with non-Hodgkin’s lymphoma (NHL) and multiple myeloma (MM) [119–121]. Moreover, it is suggested that AMD3100-mobilized cell products are associated with greater capacity to repopulate the marrow and potential of protecting against graft-versus-host disease due to an enrichment of regulatory T cells (GVHD) [118, 122]. AMD3100 has been approved for HSC mobilization and subsequent autologous transplantation in patients with NHL and MM [123]. Therefore, before the efficacy and safety of new regimens in human were well established, G-CSF in combination with AMD3100 remains the most efficient and safe regimens in patients with high risk of mobilization failure. Although G-CSF plus AMD3100 significantly improved mobilization efficiency compared with G-CSF alone, two well-designed RCTs indicated that successful rate of achieving optimal target with G-CSF plus AMD3100 is only 59.3% in NHL patients and 75.7% in MM patients [119, 120]. Therefore, we speculate that almost 25–40% of patients with high risk of mobilization failure would still benefit from new mobilization regimens.

Nevertheless, there are some limitations in this study. Firstly, we integrated evidences from animal models. It is suggested that HSC mobilization is evolutionarily conserved from mice to humans, so mice models also represent a valuable experimental system for investigating the efficacy and mechanisms of mobilization regimens [67, 81]. Even so, animal model could not completely simulate the physiological condition of human; hence, the translation of our results integrated from preclinical studies to human should be in cautions. Future clinical trials are needed for validation these regimens in human. Secondly, our meta-analysis did not include safety outcomes. Most of the included studies did not provide information about toxicity, and the toxicity data collected from animal experiments are hard to be pooled with meta-analysis. Further studies are required to compare the safety of these new mobilization regimens. Thirdly, the results of meta-analysis may be confounded by the heterogeneity in mice gender since it was reported that male mice have better mobilization outcome compared with female mice [114]. It is impractical to perform subgroup analysis based on animal gender since most of the studies did not report the gender of mice and some studies included both male and female mice. However, since the network meta-analyses were performed with well-established methods and the most efficacious regimens are associated with robust MD values, we believe that the effects of these differences are minimal. Last but not least, there is a big gap between our results and translational medicine since the lack of data from human systems, but we think this study may contribute to the translation of basic research results into clinical investigations through providing comprehensive review of new promising mobilization regimens and related mechanisms.

## Conclusions

In summary, this study identified several promising mobilization agents and regimens that significantly increased the mobilization of HSCs compared with the conventional agent G-CSF alone. We think that our results can provide important perspectives for future researches.

## Supplementary Information

**Additional file 1: Supplementary Table 1**. Characteristics of the 95 studies included for review.

**Additional file 2: Supplementary Table 2**. Risk of bias assessment using the SYRCLE tool.

**Additional file 3: Supplementary Table 3**. Results about the mobilization of different LSK subsets.

**Additional file 4: Supplementary Table 4**. Characteristics and results of in vivo transplantation experiments.

## Data Availability

All supporting data are included in the article and its additional files.

## Abbreviations

ALT1188: A small molecule CXCR4 antagonist
AMD3100: A C-X-C chemokine receptor type 4 (CXCR4) antagonist
AMP: Adenosine monophosphate
ATP: Adenosine triphosphate
BOP: A dual α9β1/α4β1 antagonist
BM: Bone marrow
CFCs: Colony-forming cells
CFUs: Colony-forming units
CASIN: Cell division control protein 42 (Cdc42) activity-specific inhibitor
CasNa: Sodium caseinate
CY: Cyclophosphamide
DFS: Disease-free survival
CWHM-823: Small molecule very late antigen 4 (VLA4) antagonist
CrI: Credibility interval
DIC: Deviance information criterion
EP80031: Synthetic octo-saccharides, a heparan sulfate mimetic
FG-4497: Hypoxia-inducible transcription factor prolyl hydroxylase domain enzymes inhibitor
FLT-3L: fms-like tyrosine kinase-3 ligand
G-CSF: Granulocyte colony-stimulating factor
HF51116: A new CXCR4 antagonist
HSCs: Hematopoietic stem cells
HSCT: Hematopoietic stem cell transplantation
HSPCs: Hematopoietic stem and progenitor cells
LD: Low dose
LFA-1: Leukocyte function antigen-1
LGB321: Proviral integration site for Moloney murine leukemia virus (PIM) kinase inhibitor
LSK cells: Lin^−^Sca1^+^ Kit^+^ cells;
LT-HSCS: Long-term hematopoietic stem cells
MD: Mean differences
Mac-1: Macrophage antigen-1
Me6: Me6TREN, Tris[2-(dimethylamino)ethyl]amine)
MIP-2: Macrophage inflammatory protein-2
ML141: Cell division control protein 42 (Cdc42) inhibitor
NA: Not applicable
Neam-r9: Nona-d-arginine-neamine conjugate
Neo-r9: Nona-d-arginine-neomycin conjugate
NOX-A12: A PEGylated mirror-image oligonucleotide that binds to CXCL12 and inhibits CXCL12 signaling
NSAIDs: Nonsteroidal anti-inflammatory drugs
OS: Overall survival
PB: Peripheral blood
PBS: Phosphate-buffered saline
PBSCs: Peripheral blood stem cells
PEGLip-G-CSF: Pegylated liposome formulated granulocyte colony-stimulating factor
POL5551: A peptidic CXCR4 antagonist
RARα: Retinoic acid receptor alpha
rhPlGF-1: Recombinant human placental growth factor-1
r9: N-α-acetyl-nona-D-arginine
SCA: Sulfated colominic acid
SD: Standard dose
SDF-1α: Stromal cell-derived factor-1α
SLAM LSK cells: Lin^−^Sca-1^+^c-kit^+^ CD48^−^CD150^+^ cells
SYRCLE: SYstematic Review Centre for Laboratory animal Experimentation
S1PR1: Sphingosine-1-phosphate receptor 1
S1PR3: Sphingosine-1-phosphate receptor 3
THI: Tetrahydroxybutylimidazole
T-140: 4F-benzoyl-TN14003, a short-modified peptide, a highly selective CXCR4 antagonist
uPAR: Urokinase receptor
VCAM-1: Vascular cell adhesion molecule-1
VLA-4: Very late antigen-4
VPC01091: A selective sphingosine-1-phosphate receptor 3 antagonist
