# Supplementary material for: Comparison of the efficacy of hematopoietic stem cell mobilization regimens: a systematic review and network meta-analysis of preclinical studies
Source: Stem Cell Res Ther. 2021 May 29;12:310. doi: 10.1186/s13287-021-02379-6 (PMC8164253; doi:10.1186/s13287-021-02379-6)
Supplement: Supplementary file 1 — Additional file 1: Supplementary Table 1. Characteristics of the 95 studies included for review. [file 13287_2021_2379_MOESM1_ESM.docx]

**Supplementary Table 1. Characteristics of the 95 studies included for review.**

| **Study** | **Mice strain** | **Sex** | **Age** | **Available outcome** | **Mobilization regimen** | **Dosage** |
| --- | --- | --- | --- | --- | --- | --- |
| Lord 1995 | BDF1 | Male | 10-12 weeks | CFU-S/L PB | MIP-1α; G-CSF; G-CSF + MIP-1α | MIP-1α: 2.5 μg/mouse s.c.; G-CSF: 100 μg/kg s.c. twice daily for 2 days |
| Mauch 1995 | C57BL/6 | Male | NA | CFU-S/10^6^ Blood Cells | IL-11; SCF; IL-11 + SCF | IL-11: 250 μg/kg s.c. once or twice daily for 7 days; SCF: 100 μg/kg s.c. twice daily for 7 days |
| Neben 1995 | C57BL/6 | Male | NA | CFU-S/10^6^ Blood Cells | CY; G-CSF; CY + G-CSF | CY: 200 mg/kg i.p. 6-7 days before harvest; G-CSF: 250 μg/kg/day s.c. twice daily for 4 days |
| Brasel 1997 | C57BL/6 | Female | 8-12 weeks | CFU-GM/ml PB, CFU-GEMM/ml PB | FLT-3L; G-CSF; GM-CSF; FLT-3L + G-CSF; FLT-3L + GM-CSF | FLT-3L: 10 μg/mouse/day s.c. for 2-10 days; G-CSF: 5 or 10 μg/mouse/day s.c. or i.p. for 2-10 days; GM-CSF: 5 or 10 μg/mouse/day s.c. or i.p. for 2-10 days |
| Sudo 1997 | C57BL/6 | NA | 5-6 weeks | Fold change of CFCs, CFCs/ml PB, CFU-GM%, BFU-E%, CFU-Mix% | FLT-3L; G-CSF; FLT-3L + G-CSF | FLT-3L: 20 μg/kg/day s.c. for 5 days; G-CSF: 250 μg/kg/day s.c. for 5 days |
| Wang 1997 | C57BL/6 | NA | 8-12 weeks | LSK cells/ml PB, CFU-GM/ml PB | MIP-2; G-CSF; G-CSF + MIP-2 | MIP-2 20 μg/kg i.v. 15 min before harvest; G-CSF: 100 μg/kg s.c. every 12 hours 2 days |
| Neap 1998 | C57BL/10 | Male | 4-6 weeks | LSK cells/ml PB | FLT-3L; G-CSF; G-CSF + FLT-3L | FLT-3L: 10 μg/day s.c. on for 10 days; G-CSF: 7.5 μg/day s.c. on day 4-10 |
| Patchen 1998 | C3H/HeN | Male and female | 7 weeks | CFU-GM/ml PB | PGG-Glucan; G-CSF; G-CSF + PGG-Glucan | G-CSF: 125 μg/kg/day s.c. for 3 days; PGG-Glucan: a single dose of 2 mg/kg i.v. |
| Torii 1998 | BDF1 | Male and female | 7-8 weeks | CFU-GM, CFU-MK, BFU-E and CFU-Mix per ml PB | PEG-MGDF; G-CSF; G-CSF + PEG-MGDF | PEG-MGDF: 100 or 300 μg/kg/day i.p. for 5 days; G-CSF: 250 or 500 μg/kg/day i.p. for 5 days |
| Zhang 1998 | BALB/c | Female | 8-12 weeks | CFU-Mix/L PB, CFU-S/L PB | IL-8; G-CSF; IL-8 + G-CSF | IL-8: 30 μg per mouse i.p. 15 min before harvest; s.c. G-CSF: 2.5 μg per mouse twice daily for 2 days |
| Verma 1999 | C57BL/6 | Female | 6-8 weeks | CFCs/10^5^ PB cells | Paclitaxel; CY | Paclitaxel: 60 mg/kg i.p. 4-6 days before harvest; CY: 200 mg/kg i.p. 4-6 day before harvest |
| de Haan 2000 | C57BL/6, DBA/2 and AKR | Female | 10-14 weeks | CAFCs/ml PB, CFU-GM per femur and per spleen | SD/0; G-CSF | SD/0: 25 μg/mouse s.c.; G-CSF: 25 μg/mouse s.c. |
| Frenette 2000 | C57BL/6 | NA | NA | CFCs per ml PB, per femur, or per spleen | Fucoidan; Heparin | Fucoidan: 2 doses of 25 mg/kg i.p.; Heparin: 2 doses of 100 U i.p. |
| Kikuta 2000 | C57BL/6 | NA | 12-14 weeks | Fold change of CFCs and CFU-S; CFCs, CFU-GM, BFU-E, CFU-Mix, and CFU-S per ml PB | Anti-VLA-4 Ab; Anti-VCAM-1 Ab; Anti-VCAM-1 Ab + G-CSF | Anti-VLA-4 Ab: 5 mg/kg i.v. for 2 days; Anti-VCAM-1 Ab: 5 mg/kg i.v. for 2 days; G-CSF: 125 μg/kg s.c. twice daily for 5 days |
| Robinson 2000 | BALB/c | Female | 6-8 weeks | CFCs/ml PB, CD34^+^ cells %, Sca-1^+^ cells %, CD34+Sca-1^+^ cells % | FLT-3L; GM-CSF; FLT-3L + GM-CSF | FLT-3L: 10 μg/mouse i.m. for 10 days; GM-CSF: 6 μg/mouse i.m. |
| Sweeney 2000 | BDF1 | NA | NA | CFCs/ml PB | Fucoidan; Linear fucan; Dextran sulfate; Chondroitin sulfate A or B; Heparan sulfate; Dextran; Hyaluronic acid; G-CSF; G-CSF + Fucoidan | G-CSF: 50 μg/kg twice daily for 3 days; Polysaccharides: 50 mg/kg i.v. once or twice |
| Fleming 2001 | C57BL/6 | Male | 8-12 weeks | CFU-S/ml PB | Progenipoietin-1; FLT-3L; G-CSF; FLT-3L + G-CSF | Progenipoietin-1: 5-100 μg/kg/day s.c. for 5 days; G-CSF: 50 or 100 μg/kg/day s.c. for 5 days; FLT-3L: 50 or 100 μg/kg/day s.c. for 5 days |
| Honda 2001 | C57BL/6 | Male and Female | 6-8 weeks | CFU-S/ml PB | PEG-rHuMGDF; G-CSF; PEG-rHuMGDF + G-CSF; | PEG-rHuMGDF: 37.5-300 μg/kg/day s.c. for 5-10 days; G-CSF: 125-500 μg/kg/day s.c. for 5 days |
| King 2001 | BDF1 | NA | 8-12 weeks | CFU-GM, CFU-G, BFU-E, CFU-GEMM, CFU-Meg per ml PB | tGROβ; G-CSF; G-CSF + tGROβ | tGROβ: 2.5mg/kg s.c. 15 min before harvest; G-CSF: 50 μg/kg s.c. twice daily for 4 days |
| Carlo 2002 | BALB/c | Female | 6-8 weeks | CFCs/ml PB | G-CSF; Defibrotide; G-CSF + Defibrotide | G-CSF: 5 or 10 μg/mouse/day i.p. for 5 days; Defibrotide: 15 mg/mouse/day i.p. for 5 days |
| Sweeney 2002 | BDF1 | NA | NA | CFCs/ml PB | Fucoidan; Fucosylated chondroitin sulfate; Chondroitin sulfates A or C | Fucoidan, Fucosylated chondroitin sulfate, Chondroitin sulfates A or C: 50 or 100 mg/kg i.v. 3 hours before harvest |
| Velders 2002 | BALB/c | Male | 8-12 weeks | CFU-GM/ml PB, CAFCs/ml PB | G-CSF; G-CSF + α-LFA-1; G-CSF + α-Mac-1; G-CSF + α-LFA-1 + α-Mac-1 | G-CSF: 5 μg per mouse i.p. for 2 days; α-LFA-1: a single dose of 100 μg i.p.; α-Mac-1: a single dose of 100 μg i.p. |
| Hofer 2003 | CBA × C57BL/10 F1 | Male | 3 months | CFU-GM/ml PB | DP + AMP; G-CSF; DP + AMP + G-CSF | DP: 2 mg/mouse s.c. once daily for 4 days; AMP: 5 mg/mouse i.p. once daily for 4 days; G-CSF: 3 μg/mouse s.c. once daily for 4 days |
| Meng 2003 | C57BL/6 | Male and Female | 6-12 weeks | CFU-GM, CFU-MK, CFU-E per 1×10^5^ PBMNCs, CD34^+^ PB cells % | IL-11; G-CSF; G-CSF + IL-11 | IL-11: 250 μg/kg/day s.c. for 7 days; G-CSF: 250 μg/kg/day s.c. for 7 days |
| Ojeifo 2003 | C57BL/6 | Female | 7-9 weeks | LSK cells/ml PB, CFCs/ml PB | G-CSF + Docetaxel; G-CSF + CY | G-CSF: 125 μg/kg s.c. twice daily for 5 days; Docetaxel: 30 mg/kg i.p.; CY: 200 mg/kg i.p. |
| Robinson 2003 | BALB/c | Female | 8-12 weeks | CFU-GM, HPP-CFC per ml PB, per femur and per spleen | FLT-3L; PG FLT-3L; G-CSF; G-CSF + FLT-3L; G-CSF + PG FLT-3L | FLT-3L: 5 μg/day i.m. for 3 days; PG FLT-3L: single dose of 15 μg i.m.; G-CSF: 6 μg/day i.m. for 4 days. |
| Liu 2004 | BALB/c | Male and Female | 8-12 weeks | CD34^+^ cells %, CFU-GM and CFU-S per 10^6^ MNCs | Anti-CD49d Ab; G-CSF; G-CSF + Anti-CD49d Ab | G-CSF: 200 μg/kg/day s.c. for 5 days; Anti-CD49d Ab: 2 mg/kg/day s.c. for 5 days |
| Nakamura 2004 | C57BL/6 | NA | 8-12 weeks | CFU-GM, CFU-G, CFU-M, CFU-GEMM per 10^6^ PB cells | s-kit; G-CSF; G-CSF + s-kit | s-kit: 200 μg/kg/day i.v. twice daily for 4 days; G-CSF: 125 μg/kg s.c. twice daily for 4 days |
| Pelus 2004 | BDF1 | NA | NA | CFU-GM/ml PB | GROβ; GROβ_T_; G-CSF; G-CSF + GROβ; G-CSF + GROβ_T_ | GROβ: 2.5 mg/kg s.c. 15 min before harvest; GROβ_T_: 2.5 mg/kg s.c. 15 min before harvest; G-CSF: 50 μg/kg/day s.c. for 4 days |
| Broxmeyer 2005 | C57BL/6, C3H/HeN and DBA/2 | NA | NA | Fold change of CFU-GM, BFU-E and CFU-GEMM | G-CSF; AMD3100; G-CSF + AMD3100 | G-CSF: 2.5 μg per mouse s.c. twice daily for 2-4 days; AMD3100: 5 mg/kg s.c. 1 hour before harvest. |
| Pelus 2005 | BALB/c | Female | 5-6 weeks | CFU-GM/ml PB | CTCE-0021; G-CSF; G-CSF + CTCE-0021 | CTCE-0021: 25 mg/kg as a single bolus i.v.; G-CSF 50 μg/kg twice daily s.c. for 4 days |
| Selleri 2006 | BALB/c | NA | 8 weeks | CFU-GM, BFU-E, and CFU-GEMM per ml PB; CD34^+^ cells/μl PB | G-CSF; uPAR84-95; G-CSF + uPAR84-95 | G-CSF: 250 μg/kg/day i.p. for 5 days; uPAR84-95: 3 mg/kg/day i.p. for 2 days |
| Abraham 2007 | C57BL/6 | Female | 7-8 weeks | CFCs, BFU-E and CFU-GEMM per ml PB | G-CSF; AMD3100; T-140; G-CSF + T-140; G-CSF + AMD3100 | G-CSF: 2.5 μg/mouse s.c. twice daily for 4 days; AMD3100 5 mg/kg s.c. 2 hours before harvest; T-140 5 mg/kg s.c. 2 hours before harvest |
| Broxmeyer 2007 | C3H/HeJ | NA | NA | Fold change of CFU-GM, BFU-E, and CFU-GEMM | MIP-1α; G-CSF; AMD3100; G-CSF + MIP-1α; AMD3100 + MIP-1α; G-CSF + AMD3100 + MIP-1α | MIP-1α: 5 μg/mouse i.p. 1 hour before harvest; G-CSF: 2.5 μg/mouse s.c. twice daily for 2 days; AMD3100: 5 mg/kg s.c. 1 hour before harvest |
| Carlo 2007 | BALB/c | Female | 6-8 weeks | CFCs/ml PB | G-CSF; rhPlGF-1; G-CSF + rhPlGF-1 | G-CSF: 10 μg/mouse/day i.p. once daily for 5-12 days; rhPlGF-1 10 μg/mouse/day i.p. for 5-12 days |
| Fukuda 2007 | BALB/c and C57BL/6 | NA | 6-8 weeks | CFU-GM, CFU-GEMM, CFU-Meg, KL and LSK cells per 2 × 10^6^ PBMCs | GROβ; GROβ_△4_; G-CSF; G-CSF + GROβ; G-CSF + GROβ_△4_ | GROβ: 2.5 mg/kg s.c. 15 min before harvest; GROβ_△4_: 2.5 mg/kg s.c. 15 min before harvest; G-CSF: 50 μg/kg/day s.c. for 4 days |
| Herbert 2007 | C57BL/6 and BALB/c | Male | 8-10 weeks | CFU-GM/ml PB, CFU-GEMM/ml PB, CFU/spleen | VTP195183; G-CSF; G-CSF + VTP195183 | VTP195183: 45 mg/kg/d gavage for 7 days; G-CSF: 125 μg/kg s.c. twice daily for 4 days |
| Kubonishi 2007 | C57BL/6 and DBA/2 | Male and female | 7-12 weeks | CFCs/ml PB | SCA; G-CSF; G-CSF + SCA | G-CSF: 125 μg/kg s.c. twice daily for 4 days; SCA: 100 mg/kg i.v. 30 min before harvest |
| Brunner 2008 | C57BL/6 | Male | 8-12 weeks | LSK cells%, CD45+CD34+ cells% in PB | PTH; G-CSF; G-CSF + PTH | PTH: 80 μg/kg/day i.p. for 6 or 14 days; G-CSF: 200 μg/kg/day i.p. for 5 days |
| Cramer 2008 | C57BL/6 | Female | 6-8 weeks | Fold change of LSK cells | PGG-Glucan; G-CSF; G-CSF + PGG-Glucan | PGG-Glucan: 9.6 mg/kg i.v.; G-CSF: 125 μg/kg/day s.c. for 4 days |
| Tjwa 2008 | NA | NA | NA | CFCs/10^6^ PB-MNC | G-CSF; G-CSF + Tenecteplase; G-CSF + Microplasmin | Tenecteplase: 100 mg/kg i.p.; Microplasmin: 100 μg/kg/day via osmotic minipumps for 5 days; G-CSF: 200 μg/kg/day s.c. for 5 days |
| Albanese 2009 | C57BL/6 | Male | 8 weeks | CFCs/ml PB for each mouse | OTR_4120_; OTR_4131_; Fucoidan; G-CSF; AMD3100; G-CSF + OTR_4120_ or OTR_4131;_ AMD3100 + OTR_4120_ or OTR_4131_ | OTR_4120_: 50 mg/kg i.p.; OTR_4131_: 50 mg/kg i.p.; Fucoidan: 50 mg/kg i.p.; G-CSF: 50 μg/kg i.p. twice daily for 2 days; AMD3100 :5 mg/kg i.p. |
| Bonig 2009 | C57BL/6 | NA | NA | CFCs/ml PB | G-CSF; AMDi; AMDb; G-CSF + AMDb | G-CSF: 100 μg/kg/day s.c. via osmotic minipumps for 9 days; AMDi: AMD3100 1 mg/day s.c. via osmotic minipumps (continuous injection) for 9 days; AMDb: AMD3100 bolus (100 μg) i.p. 1 hour before harvest |
| Dygai 2009 | CBA/CaLac | NA | 2 months | CFU-GM, CFU-E, CFU-F, MSC per 10^5^ MNCs | IMG-CSF; G-CSF | IMG-CSF: 100 μg/kg/day s.c. for 5 days or p.o. for 10 days; G-CSF: 100 μg/kg/day s.c. for 5 days |
| Huang 2009 | BALB/c | NA | NA | Fold change of CD34^+^ HSCs and CD133^+^ EPCs | TG-0054; G-CSF + TG-0054 | G-CSF: 100 μg/kg/day s.c. for 4 days; TG-0054: 50 mg/kg i.v. |
| Ramirez 2009 | C57BL/6 | NA | NA | CFU-GM/ml PB | BIO5192; G-CSF; AMD3100; AMD3100 + BIO5192; G-CSF + BIO5192; G-CSF +AMD3100; G-CSF +AMD3100 + BIO5192 | BIO5192 1mg/kg i.v.; G-CSF: 250 μg/kg/day s.c. for 5 days; AMD3100 5 mg/kg s.c. |
| Yatuv 2009 | BALB/c | Male | 8-12 weeks | Lin^-^Sca-1^+^ cells/ml PB | G-CSF; PEGLip-G-CSF | G-CSF: 300 μg/kg/day i.v. for 2 days; PEGLip-G-CSF 300 μg/kg/day i.v. for 2 days |
| de Kruijf 2010 | C57BL/6 | Male | 8-12 weeks | CFU-GM/ml PB, CFU-GM/femur, CAFC/10^5^ PBMC | FLT-3L; IL-8; FLT-3 + IL-8 | IL-8: A single dose of 30 μg i.p.; FLT-3L: 10 μg/day i.p. for 3-10 days; |
| Ryan 2010 | C57BL/6 | NA | NA | CFCs/ml PB | G-CSF; G-CSF + Erlotinib | G-CSF: 100 μg/kg/day i.p. for 5 days; Erlotinib: 2.5, 5 or 10 μg/g i.p. for 3 days |
| Tchernychev 2010 | BALB/c and DBA/2 | NA | NA | CFU-GM/10^6^ WBC | ATI-2341; AMD3100 | ATI-2341: 2 μmol/kg i.v. 1 hour before harvest; AMD3100: 2 μmol/kg i.v. 1 hour before harvest |
| Berchanski 2011 | B6.SJL | NA | 8-10 weeks | CFCs/ml PB | AMD3100; Neo-r9 + AMD3100; Neam-r9 + AMD3100; r9 + AMD3100 | AMD3100: 5 mg/kg s.c. 1-1.5 hours before harvest; Neo-r9: 1 mg/kg s.c. 1-1.5 hours before harvest; Neam-r9: 1 mg/kg s.c. 1-1.5 hours before harvest; r9: 1 mg/kg s.c. 1-1.5 hours before harvest |
| Chigaev 2011 | C57BL/6 | Male | 9-13 weeks | CFCs/ml PB | Thioridazine; AMD 3100 | Thioridazine: 1.25 mg/kg i.p. 1 hour before harvest; AMD 3100: 5 mg/kg i.p. 1 hour before harvest |
| Dar 2011 | BALB/c and C57BL/6 | NA | 8-10 weeks | CFCs per 2 × 10^5^ MNCs | Norepinephrine; AMD3100; Norepinephrine + AMD3100 | Norepinephrine: 5 mg/kg i.p.; AMD3100: 5 mg/kg s.c. |
| di Giacomo 2012 | C57BL/6 | NA | NA | CFCs, LSK cells, CFU-GEMM, CFU-GM, BFU-E, CFU-G, CFU-M per ml PB | EP80031; G-CSF + EP80031; AMD3100 + EP80031; G-CSF + AMD3100; G-CSF + AMD3100 + EP80031 | EP80031: 15 mg/kg i.v. 1 hour before harvest; G-CSF: 2.5 μg/mouse s.c. twice daily for 4 days; AMD1300: 5 mg/kg s.c. 1 hour before harvest |
| Juarez 2012 | C57BL/6 and DBA/2 | NA | NA | CFCs/ml PB for each mouse | SEW2871; G-CSF; AMD 3100; SEW2871 + G-CSF; SEW2871 + AMD 3100; G-CSF + AMD 3100; SEW2871 + G-CSF + AMD 3100 | SEW2871: 10 mg/kg s.c.; G-CSF: 125 μg/kg s.c. twice daily for 4 days; AMD 3100: 10 mg/kg s.c.; TC14012: 10 mg/kg s.c.; |
| Lucas 2012 | C57BL/6 | Male | 8 weeks | CFCs, LSKF cells, and LTC-IC per ml PB | G-CSF; G-CSF + Desipramine; AMD3100; AMD3100 + Desipramine; G-CSF + Reboxetine | G-CSF: 125 μg/kg s.c. twice daily for 4 days; AMD 3100: 5 mg/kg s.c. 1 hour before collection; Desipramine: 10 mg/kg/day i.p. for 8 days; Reboxetine: 5 mg/kg/day i.p. for 8 days |
| Chen 2013 | C57BL/6 | NA | NA | LSK cells% and KL cells% in PB | Adrenaline; G-CSF; G-CSF + Adrenaline | G-CSF: 2.5 or 5 μg/mouse/day s.c. for 5 days; Adrenaline: 50 μg/mouse/day i.p. for 5 days |
| Dygai 2013 | CBA/CaLac | Male | 2.0-2.5 months | CFU-E, CFU-GM, CFU-F and MSC per 2.5×10^5^ MNCs | Im-HD; Hyaluronidase; G-CSF | Im-HD: 1,000 U/kg/day i.p. or 50-1,000 U/kg/day p.o. for 2 days; Hyaluronidase: 1,000 U/kg/day i.p. for 2 days; G-CSF: 100 or 125 μg/kg/day s.c. for 5 days |
| Hoggatt 2013 | C57BL/6 | NA | NA | LSK, CFC, CFU-GM, BFU-E, CFU-GEMM per ml PB | Meloxicam; Indomethacin; G-CSF; AMD3100; G-CSF + Meloxicam, Indomethacin, AH23848 or L-161,982; AMD3100 + Meloxicam; G-CSF + AMD3100; G-CSF + AMD3100 + Meloxicam | G-CSF: 50 μg/kg s.c. twice daily for 4 days; AMD 3100: 5 mg/kg i.p. 1 hour before collection; Meloxicam: 0.5-12 mg/kg s.c. for 4 days; Indomethacin: 0.5-2.5 mg/kg s.c. twice daily for 4 days; AH23848: 10 μg per mouse i.p. for 4 days; L-161,982: 10 μg per mouse i.p. for 4 days |
| Karpova 2013 | C57BL/6, DBA/2 and SZT-treated C57BL/6 | NA | NA | CFCs/ml PB | POL5551; G-CSF; AMD3100; G-CSF + POL5551; G-CSF + AMD3100; CY + POL5551; CY + AMD3100; CY + G-CSF | POL5551: 5 mg/kg i.p. 2 hours before harvest; G-CSF: 100 μg/kg i.p. every 12 hours for 9 doses; AMD3100: 5 or 10 mg/kg i.p.; CY: 200 mg/kg, i.p. 8 days before harvest. |
| Kook 2013 | C57BL/6 and BALB/c | NA | NA | CFCs per 5 × 10^5^ spleen cells; LSK cells per 1 × 10^5^ WBCs | UDP-G; G-CSF; UDP-G + G-CSF; | UDP-G: 200 mg/kg/day s.c. for 6 days; G-CSF: 300 μg/kg/day s.c. for 4 days |
| Vater 2013 | C3H/HeN | Female | NA | CFCs/μl PB | G-CSF; G-CSF + AMD3100; G-CSF + NOX-A12 | G-CSF: 2.5 μg s.c. twice daily for 4 days; AMD3100: 5 mg/kg s.c.; NOX-A12: 10-50 mg/kg i.v. |
| Chen 2014 | C57BL/6 | NA | NA | CFCs, LSK cells and KL cells per ml PB | G-CSF; ML141; G-CSF + ML141 | G-CSF: 200 μg/kg/day s.c. for 5 days; ML141: 10 μg/kg/day i.p. for 5 days |
| Ghobadi 2014 | C57BL/6 | NA | NA | CFCs/ml PB | Bortezomib; G-CSF; AMD3100; G-CSF + Bortezomib; AMD3100+ Bortezomib | Bortezomib: A single dose of 0.8mg/kg i.v.; G-CSF: 250 μg/kg/day s.c. for 4 days; AMD3100: 5 mg/kg s.c. |
| He 2014 | C57BL/6 | NA | 8-10 weeks | CFCs/ml PB, LSK cells/ml PB | FLT-3L; G-CSF + AMD3100; FLT-3L + AMD3100 | FLT-3L: 350 μg/kg/day i.p. for 10 days; G-CSF: 150 μg/kg/day i.p. for 5 days; AMD3100: 5 mg/kg i.p. 1 hour before harvest |
| Saez 2014 | C57BL/6 | Male | 6-12 weeks | CFCs/ml PB | G-CSF; G-CSF + Heparin; G-CSF + Anti-VCAM-1 Ab; AMD3100; AMD3100 + Heparin | G-CSF: 125 μg/kg s.c. twice for 4 days; Heparin: 100 U i.p. 1 hour before harvest; Anti-VCAM-1 Ab: 2 mg/kg/day i.v. for 3 days; AMD3100: 5 mg/kg s.c. 1 hour before harvest |
| Zhang 2014 | C57BL/6, C3H/HeN and DBA/2 | NA | NA | CFCs, CFU-GM, BFU-E, CFU-GEMM, and HPP-CFU per 0.5 ml PB | Me6; AMD3100; G-CSF; G-CSF + AMD3100; G-CSF + Me6 | Me6: 5 mg/kg s.c. 12 hours before harvest; AMD3100: 5mg/kg s.c. 1 hour before harvest; G-CSF: 2.5 μg per mouse s.c. twice daily for 4 days |
| Forristal 2015 | C57BL/6 | Male | 9-12 weeks | CFCs, LSK cells, LSK CD48^-^ CD150^+^ cells per ml PB, and per spleen | G-CSF; G-CSF + AMD3100; G-CSF + FG-4497; G-CSF + AMD3100 + FG-4497 | G-CSF: 125 μg/kg s.c. twice daily for 4 days; AMD3100: 5 mg/kg s.c. 1 hour before harvest; FG-4497: 20 mg/kg/day i.p. for 3 days |
| Santiago 2015 | BALB/c | NA | 8-12 weeks | CFCs/ml PB | CasNa; AMD3100 | CasNa: 0.1 g i.p. once every 48 hours for 4 times; AMD3100 5 mg/kg i.p. 1 hour before harvest |
| Wysoczynski 2015 | BALB/c | NA | NA | CFU-GM/μl PB, LSK CD34- cells/μl PB | G-CSF; AMD3100; G-CSF + SnPP; AMD3100 + SnPP | G-CSF: 100 μg/kg/day s.c. for 3 or 6 days; AMD 3100: 5 mg/kg s.c. 2 hours before collection; SnPP: 30 mg/kg i.p. 1 hour before G-CSF or AMD 3100 |
| Xing 2015 | ICR | NA | 5-6 weeks | LSK cells% | HS6101; G-CSF | HS6101: 27 μg/mouse s.c.; G-CSF: 2 μg/mouse s.c. for 5 days |
| Cao 2016 | C57BL/6 | Male and Female | 7-8 weeks | LSK cells and SLAM LSK cells per ml PB | BOP; BIO5192; G-CSF; AMD3100; G-CSF + AMD3100; G-CSF + BOP; AMD3100 + BOP; AMD3100 + BIO5192; G-CSF + AMD3100 + BOP | G-CSF: 250 μg/kg s.c. twice daily for 4 days; AMD 3100: 3 mg/kg s.c. 1 hour before harvest; BOP: 10 mg/kg s.c. 1 hour before harvest; BIO5192: 1 mg/kg s.c. 1 hour before harvest |
| Lu 2016 | C57BL/6 and C3H/HeJ | Male | 6-8 weeks | LSK cells/ml PB, CFCs/ml PB | LECT2; G-CSF; AMD3100; G-CSF + LECT2; AMD3100 + LECT2 | LECT2: 300 μg/kg/day s.c. for 5 days; G-CSF: 300 μg/kg/day s.c. for 5 days; AMD 3100: 5 mg/kg s.c. 1 hour before collection |
| Yan 2016 | C57BL/6 | Male and Female | 8-12 weeks | CD34^+^% in PB, CFU-Mix per 10^5^ PBMNCs | Dexamethasone; AMD3100; Dexamethasone + AMD3100 | Dexamethasone: 0.2 mg/kg s.c.; AMD3100: 5 mg/kg s.c. |
| Adamiak 2017 | C57BL/6 | Female | 6-8 weeks | LSK cells, Sca-1+ CD45+ Lin− cells and CFU-GM per μl PB | G-CSF; AMD3100; G-CSF + THI; AMD3100 + THI; G-CSF + SLM6031434; AMD3100 + SLM6031434 | G-CSF: 100 μg/kg/day s.c. for 3 days; AMD 3100: 5 mg/kg i.p. 1 hour before collection; THI: 25 mg/L administered ad libitum in water; SLM6031434: 5 mg/kg i.p. |
| Karpova 2017 | C57BL/6 | NA | NA | CFCs, LSK cells and SLAM LSK cells per ml PB | POL5551; AMD3100; ALT1188; POL5551 + AMD3100; POL5551 + CWHM-823 | POL5551: 100mg/kg i.p. as a single or as a continuous infusion for 2 weeks via subcutaneously implanted pumps; ALT1188: 33 mg/kg i.p. as a single injection or as continuous infusion for 2 weeks; AMD3100: 20 mg/kg i.p. as a single injection or as continuous infusion for 2 weeks; CWHM-823: 3 mg/kg i.p. |
| Nowlan 2017 | Humanized NSG | Female | 6-8 weeks | Lin^-^CD34^+^ cell, Lin^-^CD34^+^CD38^-^ cells per ml PB or per spleen | FG-4497; G-CSF; FG-4497 + G-CSF | FG-4497: 20 mg/kg/day i.p. for 3 days; G-CSF: 125 μg/kg s.c. twice daily for 2 or 4 days |
| Ogle 2017 | C57BL/6 | Male | 8-12 weeks | CFCs/100 μl PB, Fold change of LSK cells | VPC01091; AMD3100; AMD3100 + VPC01091 | AMD3100 5mg/kg i.p. 1.5 hours before harvest; VPC01091 5mg/kg i.p. 1.5 hours before harvest |
| Redpath 2017 | BALB/c | Female | 8-12 weeks | CFU-HPCs/ml PB | KRH3955; AMD 3100; KRH3955 + AMD 3100 | KRH3955: 30 mg/kg oral gavage 2 hours before collection; AMD 3100: 5 mg/kg i.p. 1 hour before collection |
| Wang 2017 | C57BL/6 | NA | NA | LSK cells and KL cells per ml PB for each mouse | Anti-Notch2 Ab; G-CSF; AMD3100; G-CSF + Anti-Notch2 Ab; AMD3100 + Anti-Notch2 Ab; G-CSF + AMD3100; G-CSF + AMD3100 + Anti-Notch2 Ab | G-CSF: 2.5 μg per mouse s.c. for twice daily for 2 days; AMD3100: 5 mg/kg s.c. 1 hour before harvest; Anti-Notch2 Ab: 25 mg/ml i.p. as a single dose or twice weekly three days apart for a total of 4 doses |
| Adamiak 2018 | C57BL/6 | NA | 4-6 weeks | LSK cells, Sca-1+ CD45+ Lin− cells and CFU-GM per μl PB | G-CSF; AMD3100; G-CSF + ATP; AMD3100 + ATP | G-CSF: 100 μg/kg/day s.c. for 3 days; AMD 3100: 5 mg/kg i.p. 1 hour before collection; ATP: 15 mg/kg/day for 3 days |
| Ghobadi 2018 | DBA and BALB/c | NA | NA | CFCs/ml PB | G-CSF; AMD3100; Ixazomib; G-CSF + Ixazomib; AMD3100 + Ixazomib | G-CSF: 250 μg/kg/day s.c. for 4 days; AMD3100: 5mg/kg s.c.; Ixazomib: 8 mg/kg oral gavage |
| Hoggatt 2018 | BALB/c, C57BL/6, DBA/2 and BDF1 | Male and female | 6-8 weeks old | CFU-GM/ml PB | tGROβ; AMD3100; G-CSF; AMD3100 + tGROβ | tGROβ: 2.5 mg/kg s.c. 15 min before harvest; AMD3100: 5 mg/kg s.c. 1 hour before harvest; G-CSF 62.5 μg/kg s.c. twice daily for 4 days |
| Notario 2018 | HuCD69 | NA | NA | Number of total cells in BM, PB and spleen | Anti-CD69 Ab; AMD3100 | Anti-CD69 Ab: 500 μg/mouse i.v. 24 hours before harvest; AMD3100: 150 μg/mouse i.p. 1 hour before harvest |
| Wu 2018 | C57BL/6 | Male | 8-10 weeks | CFU-GM/ml PB | CX0714; G-CSF; AMD 3100; G-CSF + AMD 3100; G-CSF + CX0714 | CX0714: 35 mg/kg s.c. 2 hours before collection; G-CSF: 100 μg/kg/day s.c. for 5 days; AMD 3100: 6 mg/kg s.c. 2 hours before collection |
| Adamiak 2019 | C57BL/6 | NA | 4-6 weeks | LSK/μl PB, CFU-GM/μl PB | G-CSF; AMD3100; G-CSF + ARL67156; AMD3100 + ARL67156; G-CSF + AMPCP; AMD3100 + AMPCP | G-CSF: 100 μg/kg/day s.c. for 3 days; AMD 3100: 5 mg/kg i.p. 1 hour before collection; ARL67156: 2 mg/kg i.p.; AMPCP: 4 mg/kg i.p. |
| Alt 2019 | C57BL/6 | Male | 6-10 weeks | LSK/ml PB; CFU-GM, BFU-E and CFU-GEMM per 10^5^ cells | G-CSF; AMD3100; G-CSF + AMD3100; IL-33; G-CSF + IL-33; AMD 3100 + IL-33; G-CSF + AMD3100 + IL-33 | G-CSF: 200 μg/kg/day s.c. for 3 days; AMD 3100: 5 mg/kg i.p. 1 hour before collection; IL-33: 0.04 mg/kg/day i.p. for 3 days |
| Bisht 2019 | C57BL/6 | Male | 8-9 weeks | CFCs, LSK cells, SLAM LSK cells per ml PB, and per spleen | G-CSF; G-CSF + FG-4497 | G-CSF: 125 μg/kg s.c. twice daily for 2 days; FG-4497: 20 mg/kg/day i.p. for 3 days; |
| Karpova 2019 | DBA2/J | Male | 6-12 weeks | CFCs/ml PB, LSK cells/ml PB | tGROβ; Firategrast; BIO5192; CWHM-823; CWHM-842; Firategrast + tGROβ; BIO5192 + tGROβ; CWHM-823 + tGROβ; CWHM-842 + tGROβ; GROα; IL-8; GROα + CWHM-823; IL-8 + CWHM-823; | tGROβ: 2 or 2.5 mg/kg s.c.; Firategrast: 100 mg/kg i.v.; BIO5192: 3 mg/kg i.v.; CWHM-823: 3 mg/kg i.v. or s.c.; CWHM-842: 3 mg/kg i.v.; GROα: 1 mg/kg s.c.; IL-8: 1 mg/kg s.c. |
| Liu 2019 | C57BL/6 | NA | NA | CFCs/ml PB | CASIN; AMD3100; G-CSF; CASIN + AMD3100; CASIN + G-CSF | CASIN: 1.2mg/kg i.v. 2 hours before harvest; AMD3100: 5mg/kg i.p. 2 hours before harvest; G-CSF: 100 μg/kg/day i.p. for 5 days |
| Muller 2019 | C57BL/6 and FvB/N | NA | NA | LSK cells/ml PB | AMD3100; AMD3100 + LGB321 | AMD3100: 5 mg/kg s.c.; LGB321: 100 mg/kg s.c. |
| Smith-Berdan 2019 | C57BL/6 | Male and Female | 8-16 weeks | LSK CD27^+^FLK2^-^ cells in PB per mouse | G-CSF; AMD3100; Viagra; AMD3100 + Viagra | G-CSF: 250 μg/kg/day s.c. for 4 days; Viagra: 3 mg/kg oral gavage 2 hours before harvest; AMD 3100: 2.5 mg/kg s.c. 1 hour before harvest |
| Szade 2019 | C57BL/6 × FVB | NA | NA | LSK CD48^-^CD150^+^cells, LSK CD48^-^CD150^-^cells, LSK CD48^+^150^-^cells; LSK CD48^+^150^+^cells per μl PB | G-CSF; CoPP | G-CSF: 250 μg/kg/day i.p. for 5 days; CoPP: 10 mg/kg i.p. for 5 days |
| Fang 2021 | C57BL/6, C3H/HEJ and DBA/2 | NA | NA | CFCs/ml PB, LSK cells//ml PB, SLAM LSK cells/ml PB | HF51116; AMD 3100; G-CSF; G-CSF + HF51116; G-CSF + AMD 3100 | G-CSF: 100 μg/kg every 12h s.c. for 4 days; AMD 3100: 5 mg/kg s.c.; HF51116:5 mg/kg s.c. |
| Kaur 2021 | C57BL/6 | Female | 10 weeks | CD48^-^CD150^+^ LSK, CD48^-^CD150^-^ MPP, CD48^+^ HPP per ml PB, per femur and per spleen | G-CSF; G-CSF + CSF1-Fc | CSF1-Fc: 1 mg/kg s.c. for 4 days; G-CSF: 125 μg/kg i.p. twice daily for 3 days |
