# Supplementary material for: Comparison of the efficacy of hematopoietic stem cell mobilization regimens: a systematic review and network meta-analysis of preclinical studies
Source: Stem Cell Res Ther. 2021 May 29;12:310. doi: 10.1186/s13287-021-02379-6 (PMC8164253; doi:10.1186/s13287-021-02379-6)
Supplement: Supplementary file 2 — Additional file 2: Supplementary Table 2. Risk of bias assessment using the SYRCLE tool. [file 13287_2021_2379_MOESM2_ESM.docx]

**Supplementary Table 2. Risk of bias assessment using the SYRCLE tool.**

| **Study** | **Random sequence generation** | **Similar baseline characteristics** | **Allocation concealment** | **Random housing** | **Blinding of caregivers and investigators** | **Random selection for outcome assessment** | **Blinding of outcome assessor** | **Adequate addressing of incomplete outcome data** | **Free from selective outcome reporting** | **Free from other bias** |
| --- | --- | --- | --- | --- | --- | --- | --- | --- | --- | --- |
| Abraham 2007 | Unclear | Yes | Unclear | Unclear | Unclear | Unclear | Unclear | Yes | Yes | Yes |
| Kubonishi 2007 | Unclear | Yes | Unclear | Unclear | Unclear | Unclear | Unclear | Yes | Yes | Yes |
| di Giacomo 2012 | Unclear | Unclear | Unclear | Unclear | Unclear | Unclear | Unclear | Yes | Yes | Yes |
| Lucas 2012 | Unclear | Yes | Unclear | Unclear | Unclear | Unclear | Unclear | Yes | Yes | Yes |
| Hoggatt 2013 | Unclear | Unclear | Unclear | Unclear | Unclear | Unclear | Unclear | Yes | Yes | Yes |
| Karpova 2013 | Unclear | Unclear | Unclear | Unclear | Unclear | Unclear | Unclear | Yes | Yes | Yes |
| Chen 2014 | Unclear | Unclear | Unclear | Unclear | Unclear | Unclear | Unclear | Yes | Yes | Yes |
| Ghobadi 2014 | Unclear | Unclear | Unclear | Unclear | Unclear | Unclear | Unclear | Yes | Yes | Yes |
| He 2014 | Unclear | Unclear | Unclear | Unclear | Unclear | Unclear | Unclear | No | Yes | Yes |
| Saez 2014 | Unclear | Yes | Unclear | Unclear | Unclear | Unclear | Unclear | Yes | Yes | Yes |
| Zhang 2014 | Unclear | Unclear | Unclear | Unclear | Unclear | Unclear | Unclear | Yes | Yes | Yes |
| Forristal 2015 | Unclear | Yes | Unclear | Unclear | Unclear | Unclear | Unclear | Yes | Yes | Yes |
| Lu 2016 | Unclear | Yes | Unclear | Unclear | Unclear | Unclear | Unclear | Yes | Yes | Yes |
| Karpova 2017 | Unclear | Unclear | Unclear | Unclear | Unclear | Unclear | Unclear | Yes | Yes | Yes |
| Ogle 2017 | Unclear | Yes | Unclear | Unclear | Unclear | Unclear | Unclear | Yes | Yes | Yes |
| Adamiak 2019 | Unclear | Yes | Unclear | Unclear | Unclear | Unclear | Unclear | Yes | Yes | Yes |
| Alt 2019 | Unclear | Yes | Unclear | Unclear | Unclear | Unclear | Unclear | Yes | Yes | Yes |
| Bisht 2019 | Unclear | Yes | Unclear | Unclear | Unclear | Unclear | Unclear | Yes | Yes | Yes |
| Liu 2019 | Unclear | Unclear | Unclear | Unclear | Unclear | Unclear | Unclear | No | Yes | Yes |
| Muller 2019 | Unclear | Unclear | Unclear | Unclear | Unclear | Unclear | Unclear | Yes | Yes | Yes |
| Fang 2021 | Unclear | Unclear | Unclear | Unclear | Unclear | Unclear | Unclear | Yes | Yes | Yes |
