# Supplementary material for: Comparison of the efficacy of hematopoietic stem cell mobilization regimens: a systematic review and network meta-analysis of preclinical studies
Source: Stem Cell Res Ther. 2021 May 29;12:310. doi: 10.1186/s13287-021-02379-6 (PMC8164253; doi:10.1186/s13287-021-02379-6)
Supplement: Supplementary file 3 — Additional file 3: Supplementary Table 3. Results about the mobilization of different LSK subsets. [file 13287_2021_2379_MOESM3_ESM.docx]

**Supplementary Table 3. Results about the mobilization of different LSK subsets.**

| **Study** | **Experimental arm** | **LSK subsets** | **Results** |
| --- | --- | --- | --- |
| Lucas 2012 | G-CSF; G-CSF + Desipramine | Lin^-^Sca-1^+^c-kit^+^Flt3^-^ (LSKF) cells | G-CSF + Desipramine significantly increased the mobilization of LSKF cells compared with G-CSF alone. |
| Hoggatt 2013 | G-CSF; G-CSF + Meloxicam | Lin^-^Sca-1^+^c-kit^+^ CD48^-^CD150^+^ (SLAM LSK) cells | G-CSF + Meloxicam significantly increased the mobilization of SLAM LSK cells compared with G-CSF alone. |
| Forristal 2015 | G-CSF; G-CSF + PHI-1; G-CSF + PHI-2 | SLAM LSK cells | Combinations of either PHI-1 or PHI-2 with G-CSF significantly increased the mobilization of SLAM LSK cells compared with G-CSF alone. |
| Cao 2016 | BOP; AMD3100; AMD3100 + BOP; BIO5192; AMD3100 + BIO5192; G-CSF; G-CSF + AMD3100; G-CSF + BOP; G-CSF + AMD3100 + BOP | SLAM LSK cells | Combinations of BOP with G-CSF or AMD3100 significantly increased the mobilization of SLAM LSK cells compared with G-CSF or AMD3100 alone. |
| Karpova 2017 | POL5551; ALT1188; AMD3100; POL5551 + AMD3100; POL5551 + CWHM-823 | SLAM LSK cells | Continuous infusion of POL5551 for 2 weeks increased the mobilization of SLAM LSK cells compared with single dose of POL5551 or AMD3100. Combinations of POL5551 with AMD3100 did not further increase mobilization. |
| Hoggatt 2018 | G-CSF; tGROβ + AMD3100 | SLAM LSK cells | tGROβ + AMD3100 mobilized higher-number and more competitive SLAM LSK cells than G-CSF. |
| Bisht 2019 | G-CSF; G-CSF + FG-4497 | SLAM LSK cells | G-CSF + FG-4497 significantly increased the mobilization of SLAM LSK cells compared with G-CSF alone. |
| Liu 2019 | CASIN; AMD3100 | LT-HSCs (Lin^-^Sca1^+^c-Kit^+^CD150^+^CD48^-^CD41^-^ cells) | CASIN induced the mobilization of LT-HSC with higher levels than those of AMD3100. |
| Smith-Berdan 2019 | AMD3100; Viagra; AMD3100 + Viagra; G-CSF + Viagra | LT-HSCs (Lin^-^Sca1^+^c-Kit^+^CD27^+^FLK2^-^ cells) | Combinations of Viagra with G-CSF or AMD3100 significantly increased the mobilization of LT-HSCs compared with G-CSF or AMD3100 alone. |
| Szade 2019 | G-CSF; CoPP | LSK CD48^-^CD150^+^ cells, LSK CD48^-^CD150^-^ cells, LSK CD48^+^150^-^ cells, LSK CD48^+^150^+^ cells and LT-HSCs (LSK CD48^-^CD150^+^CD34^-^ cells) | CoPP induced higher-levels mobilization of each LSK subset compared with G-CSF. |
| Fang 2021 | G-CSF; G-CSF + HF51116; G-CSF + AMD 3100 | SLAM LSK cells | G-CSF + HF51116 significantly increased the mobilization of SLAM LSK cells compared with G-CSF alone. |
| Kaur 2021 | G-CSF; G-CSF + CSF1-Fc | LSK CD48^-^CD150^+^cells, LSK CD48^-^CD150^-^cells and LSK CD48^+^ cells | G-CSF + CSF1-Fc significantly increased the mobilization of each LSK subset compared with G-CSF alone. |

Abbreviations: ALT1188, a small molecule CXCR4 antagonist; BOP, a dual α9β1/α4β1 integrin antagonist; BIO5192, a very late antigen-4 (VLA-4) antagonist; CASIN, cell division control protein 42 (Cdc42) activity-specific inhibitor; CoPP, Cobalt protoporphyrin IX ; CSF1-Fc, Colony-stimulating factor 1 (CSF1) Fc fusion protein; CWHM-823, aVLA-4 antagonist; FG-4497: hypoxia-inducible transcription factor prolyl hydroxylase domain (PHD) enzymes inhibitor; G-CSF, granulocyte colony-stimulating factor; HF51116, a new CXCR4 antagonist; LSK cells, Lin^-^Sca1^+^ Kit^+^ cells; LT-HSCS, long-term hematopoietic stem cells; PHI-1, PHD inhibitor; PHI-2, PHD inhibitor; POL5551, a peptidic CXCR4 antagonist; SLAM, signalling lymphocyte activation molecule; SLAM LSK cells, Lin^-^Sca-1^+^c-kit^+^ CD48^-^CD150^+^ cells.
