# Supplementary material for: Comparison of the efficacy of hematopoietic stem cell mobilization regimens: a systematic review and network meta-analysis of preclinical studies
Source: Stem Cell Res Ther. 2021 May 29;12:310. doi: 10.1186/s13287-021-02379-6 (PMC8164253; doi:10.1186/s13287-021-02379-6)
Supplement: Supplementary file 4 — Additional file 4: Supplementary Table 4. Characteristics and results of in vivo transplantation experiments. [file 13287_2021_2379_MOESM4_ESM.docx]

**Supplementary Table 4. Characteristics and results of *in vivo* transplantation experiments.**

| **Study** | **Mobilization regimen** | **Recipient mice** | **Donor mice** | **Irradiation dose for recipients** | **Donor cells dose** | **Competitor cells** | **Secondary transplant cells** | **Primary Outcome** | **Results** |
| --- | --- | --- | --- | --- | --- | --- | --- | --- | --- |
| Mauch 1995 | IL-11; SCF; IL-11 + SCF | B6-Hbb^s^ | B6-Hbb^s^ | 12.5 Gy | 2-10 × 10^5^ PB or BM cells | 5 × 10^5^ BM cells from B6-Hbb^d^ mice | NA | CRI at 6 months | IL-11 or SCF alone did not increase blood LTRA; IL-11 + SCF enhanced the mobilization of long-term marrow-repopulating cells. |
| Neben 1995 | CY; G-CSF; CY + G-CSF | B6-Hbb^s^ | B6-Hbb^s^ | 12.5 Gy | 2-10 × 10^5^ PB or BM cells | 5 × 10^5^ BM cells from B6-Hbb^d^ mice | NA | CRI at 6 months | Blood mobilized with CY, G-CSF, and CY + G-CSF demonstrated improved LTRC numerically approaching that of normal BM. |
| Brasel 1997 | FLT-3L; FLT-3L + G-CSF | C57BL/6-Ly5.1 | C57BL/6-Ly5.2 | 10 Gy | 1-5 × 10^5^ PBMCs | NA | NA | Survival % for 30 days; Short-term (30 days) and Long-term (6 months) reconstitution | FLT-3L- or FLT-3L + G-CSF-mobilized PB cells showed great survival and good short-term/long-term reconstitution. |
| Lord 1995 | MIP-1α; G-CSF; MIP-1α + G-CSF | BDF1 | BDF1 | 15.25 Gy | 0.2 ml PB | NA | BM from primary recipients | MRA | MIP-1α + G-CSF enhanced the mobilization of cells with MRA compared with G-CSF alone. |
| Neipp 1998 | FLT-3L; G-CSF; G-CSF + FLT-3L | B10 | B10.BR | 9.5 Gy | 1-5 × 10^6^ PBMCs | NA | NA | Survival % for 6 months; Short-term (30 days) and Long-term (6 months) engraftment | FLT-3L- or FLT-3L + G-CSF-mobilized PB cells showed great survival and good short-term/long-term reconstitution. |
| Patchen 1998 | PGG-Glucan; G-CSF | C3H/HeN female mice | C3H/HeN male mice | 8.5 Gy | 3.4 × 10^6^ PBMC from male mice | 3.4 × 10^6^ PBMC from female mice | NA | Short-term (2 weeks) and Long-term (3 months) reconstitution | PGG-Glucan-mobilized PB cells showed comparable reconstitution that of G-CSF. |
| Torii 1998 | PEG-MGDF | BDF1 female mice | BDF1 male mice | 11 Gy | 250 μl PB | NA | NA | Survival % for 200 days; Long-term (6 months) engraftment | PEG-MGDF-mobilized PB cells showed great survival and significant donor engraftment. |
| Zhang 1998 | IL-8; G-CSF; IL-8 + G-CSF | BALB/c | BALB/c | 7.5 Gy | 2 × 10^6^ PBMNCs | NA | 5 × 10^5^ BMMNCs from primary recipients | MRA | IL-8 alone or in combination with G-CSF significantly increase the number of cells with MRA in PB. |
| Frenette 2000 | Fucoidan | C57BL/6-Ly5.2 (CD45.1^+^) | C57BL/6-Ly5.1 (CD45.2^+^) | 12 Gy | 1 ml PB | 1 × 10^5^ BM cells from CD45.1+ mice | NA | CRU for 1-6 months | Fucoidan-mobilized PB cells showed great competitive repopulating ability. |
| Sweeney 2000 | Fucoidan | BDF1 | BDF1 | 12 Gy | 0.131 ml PB | NA | NA | Survival % for 120 days | All mice injected with Fucoidan-mobilized blood survived more than 120 days. |
| Fleming 2001 | ProGP; G-CSF; FLT-3L + G-CSF | C57BL/6-Ly5.1 | B6.SJL-Ly5.2 | 11 Gy | 1-100 μl PB | 2 × 10^5^ Ly5.1 BM cells | 1-10 × 10^6^ BM cells from primary recipients | Survival% for 50 days; Long-term (6 months) multilineage reconstitution | ProGP-mobilized PB cells provide radioprotection, long-term multilineage reconstitution and serial transplantation capacity. |
| Honda 2001 | PEG-rHuMGDF; G-CSF; PEG-rHuMGDF + G-CSF; | C57BL/6 female | C57BL/6 male | 8 Gy | 300 μl PB | NA | NA | Long-term (4 months) donor-derived engraftment (Y-chromosome positivity) | All recipients transplanted with PBPCs mobilized by PEG-rHuMGDF, G-CSF or PEG-rHuMGDF + G-CSF showed long-term donor type reconstitution |
| King 2001 | SB-251353; G-CSF; SB-251353 + G-CSF | BDF1 female | BDF1 male | 12.5 Gy | 1 × 10^6^ PB cells | NA | NA | Survival% and Y chromosome positivity for 100 days | SB-251353 alone or in combination with G-CSF mobilizes HSCs with long-term repopulating ability. |
| Crarlo 2002 | G-CSF; Defibrotide + G-CSF | BALB/c | BALB/c | 10.5 Gy | 2-5 × 10^5^ PBMNCs | NA | NA | Survival % for 33 days | Recipients of PBMNCs mobilized by Defibrotide + G-CSF showed greater survival than that of G-CSF alone. |
| Liu 2004 | Anti-CD49d Ab; G-CSF; G-CSF + Anti-CD49d Ab | BALB/c | BALB/c | 7.5 Gy | 2 × 10^6^ PBMNCs | NA | NA | Survival % for 4 weeks | Recipients of PBMNCs mobilized by G-CSF + Anti-CD49d Ab showed greater survival than that of G-CSF alone. |
| Nakamura 2004 | G-CSF; G-CSF + s-kit | C57BL/6-Ly5.2 | C57BL/6-Ly5.1 | 9 Gy | 3.5 × 10^4^ or 3.5 × 10^5^ Lin^-^ PB cells | 2 × 10^5^ Ly5.2 BM cells | NA | Donor cells % and CRU at 6 months | PB cells mobilized by G-CSF + s-kit showed greater long-term multilineage engraftment than that of G-CSF alone. |
| Broxmeyer 2005 | G-CSF; AMD3100; G-CSF + AMD3100 | B6.BoyJ (CD45.1^+^) | C57BL/6 (CD45.2^+^) | 9.5 Gy | 0.5-1.5 × 10^6^ PB LDMNCs | 0.5 × 10^6^ CD45.1+ BM cells | 2.5 × 10^6^ BM cells from primary recipients | Long-term (6 months) donor-derived reconstitution | AMD3100 synergized with G-CSF to mobilize LTR, self-renewing HSCs that engrafted primary and secondary recipients. |
| Abraham 2007 | AMD3100; T-140 | C57BL/6 | C57BL/6 | 9 Gy | 225 or 900 μl PB | NA | BM cells from primary recipients | Survival % for 50 days | T-140 mobilizes HSCs with long-term repopulating capacity that can produce long-term rescue in lethally irradiated primary and secondary transplanted mice. |
| Crarlo 2007 | G-CSF; rhPlGF-1; G-CSF + rhPlGF-1 | BALB/c | BALB/c | 8.5 Gy | 2 or 5 × 10^5^ PBMNCs | NA | NA | Survival % for 60 days | rhPlGF-1 + rhG-CSF-mobilized cells resulted in higher survival than that of G-CSF alone. |
| Fukuda 2007 | GROβ_△4_; G-CSF; G-CSF + GROβ_△4_ | B6.BoyJ (CD45.1^+^) | C57BL/6 (CD45.2^+^) | 10.5 Gy | 1-2 × 10^6^ PBMCs | 0.5 × 10^6^ CD45.1^+^ BM cells | 1 × 10^6^ BM cells from primary recipients | Donor chimerism % at 6 months | GROβ_△4_ or G-CSF + GROβ_△4_-mobilized cells showed enhanced donor chimerism in primary, secondary and tertiary recipients |
| Herbert 2007 | VTP195183; G-CSF; G-CSF + VTP195183 | B6.BoyJ (CD45.1^+^) | C57BL/6 (CD45.2^+^) | 10 Gy | 5 × 10^5^ PB leukocytes | 2 × 10^5^ CD45.1^+^/CD45.2^+^ BM cells | NA | RU/mL PB 5 weeks, 4 months and 6 months | VTP195183 + G-CSF-mobilized HSCs showed enhanced long-term repopulation potentials in competitive transplant assay. |
| Kubonishi 2007 | G-CSF + SCA; G-CSF | C57BL/6 (CD45.1^+^) | G-CSF + SCA-treated C57BL/6 (CD45.2^+^) | 13 Gy | 150 μl PB | 150 μl PB from G-CSF-treated CD45.1^+^ mice | NA | Donor chimerism % for 4-24 weeks | 2-day G-CSF plus single-dose SCA mobilizes long-term reconstituting HSCs as efficient as 4-day G-CSF. |
| Cramer 2008 | PGG-Glucan; G-CSF; G-CSF + PGG-Glucan | B6.SJL (CD45.1^+^) | C57BL/6 (CD45.2^+^) | 8 Gy | 500 LSK cells | NA | NA | CD45.2^+^ donor cells engraftment on day 30 | PGG-Glucan-mobilized cells had similar levels of engraftment compared with those mobilized by G-CSF. |
| Albanese 2009 | OTR4120; OTR4131; Fucoidan | C57BL/6 (CD45.1^+^) | C57BL/6 (CD45.2^+^) | 9 Gy | 4 × 10^6^ PBMCs | NA | NA | Survival% and CD45.2^+^ chimerism at 150 days | OTR4131 mobilizes HSCs that are able to radioprotect engrafted mice from lethal irradiation and to reconstitute the recipient hematopoiesis, but less efficient than Fucoidan. |
| Ramirez 2009 | BIO5192; G-CSF; AMD3100; AMD3100 + BIO5192 | C57BL/6 × B6.SJL F1 (CD45.1/CD45.2) | C57BL/6 (CD45.2^+^) | 9 Gy | 3.0-3.5 ml PB | 0.5 × 10^6^ CD45.1^+^ BM cells | 1 × 10^6^ BM cells from primary recipients | CD45.2^+^ donor cells engraftment for 1-5 months | Engraftment of mice that received HSPCs mobilized by single-dose AMD3100 and BIO5192 approached that of a 4-day G-CSF. |
| de Kruijf 2010 | G-CSF; FLT-3L; FLT-3L + IL-8 | C57BL/6-Ly5.1 or BALB/c | C57BL/6-Ly5.2 or BALB/c | 9.5 Gy | 1.5 × 10^6^ PBMCs | NA | NA | Survival% for 250 days; Long-term (27 weeks) multilineage donor chimerism | 10-day FLT-3L induces the mobilization of HSCs with long-term repopulating ability comparable to that of G-CSF; 5-day FLT-3L + IL-8 induces the mobilization of radioprotective HSC. |
| Ryan 2010 | G-CSF; G-CSF + Erlotinib | B6.SJL(CD45.1^+^) | C57BL/6 (CD45.2^+^) | 11.75 Gy | 2 × 10^6^ PB cells | 2 × 10^6^ CD45.1^+^ BM cells | NA | RU at 3 months | G-CSF + Erlotinib increased mobilization of HSCs with higher RU value in competitive transplantation. |
| di Giacomo 2012 | G-CSF + EP80031; G-CSF + AMD3100; G-CSF + AMD3100 + EP80031 | C57BL/6 (CD45.2^+^) | C57BL/6 (CD45.1^+^) | 11 Gy | 15000 PB KLS cells | 3000 BM KLS cells from CD45.2^+^ mice | 50000 BM cells from primary recipients | Long-term (4 months) CD45.1 chimerism | Animals received PB KLS cells mobilized with G-CSF + AMD3100 or G-CSF + AMD3100 + EP80031 showed the greatest chimerism compared with G-CSF alone. |
| Juarez 2012 | SEW2871 + AMD3100; AMD3100 | B6.SJL (CD45.1^+^) | C57BL/6 (CD45.2^+^) | 11 Gy | 500 μl PB | 0.25 × 10^6^ BM cells from RFP mice | NA | Donor (CD45.2^+^RFP^-^) chimerism for 5-15 weeks | SEW2871 + AMD3100-mobilized HSCs resulted in higher chimerism compared with AMD3100 alone. |
| Hoggatt 2013 | G-CSF; G-CSF + Meloxicam | C57BL/6 (CD45.2^+^) | B6.SJL (CD45.1^+^) | Lethally irradiated | 5-20 × 10^5^ PBMCs | 2 or 5 × 10^5^ CD45.2^+^ BM cells | BM cells from primary recipients | Chimerism, CRU and multilineage reconstitution for 12, 24 and 36 weeks. | Staggered G-CSF + Meloxicam mobilization resulted in enhancement of LT-HSC activity with multi-lineage reconstitution. |
| Karpova 2013 | POL5551; G-CSF | C57BL/6 (CD45.2^+^) | B6.SJL (CD45.1^+^) | 9.5 Gy | 2.5-10 μl PB | 2.5 ×10^5^ CD45.2^+^ BM cells. | NA | CRU at 16 weeks | POL5551 and G-CSF mobilized CRU into blood at similar frequencies. |
| Kook 2013 | UDP-G; G-CSF; UDP-G + G-CSF | C57BL/6 CD45.1/CD45.2 mice | UDP-G-treated CD45.2 mice | 9.5-10 Gy | 2 × 10^6^ PB cells | PB cells from G-CSF-treated CD45.1 mice | BM cells from primary recipients | Donor chimerism for 1-5 months | UDP-G-mobilized cells display superior long-term repopulating capacity compared with G-CSF-mobilized cells; UDP-G + G-CSF has an improved the mobilization of HSCs with LTRC over G-CSF alone. |
| He 2014 | FLT-3L; G-CSF; AMD3100; G-CSF + AMD3100; FLT-3L + AMD3100 | C57BL/6 or BALB/c | C57BL/6 | 9 Gy | 2 or 8 × 10^5^ PB cells | NA | NA | Survival % at 4 months | The engraftment of the cells mobilized by FLT-3L + AMD3100 was significantly superior to that of the cells mobilized by G-CSF + AMD3100. |
| Saez 2014 | G-CSF; G-CSF + Heparin; G-CSF + Anti-VCAM-1 Ab | B6.SJL (CD45.1^+^) | C57BL/6 (CD45.2^+^) | 9.5 Gy | 3 × 10^6^ PBMCs | 2.5 × 10^5^ CD45.1^+^ BM cells | BM cells from primary recipients | Donor chimerism for 6-16 weeks | G-CSF + Anti-VCAM-1 Ab increased donor chimerism compared with G-CSF alone; G-CSF + Heparin increased the mobilization of long-term reconstituting and efficiently self-renewed cells as measured by competitive transplantation and secondary transplantation. |
| Zhang 2014 | Me6; AMD3100 | C57BL/6 (CD45.1) | C57BL/6 (CD45.2) | 8-9.5 Gy | MNCs from 2 mL PB | 5 × 10^5^ CD45.1^+^ BM cells | 5×10^6^ BM MNCs from primary recipients | Survival % for 120 days; Long-term (6 months) multilineage reconstitution | Me6-mobilized HSPCs showed more effective long-term rescue of irradiated donors, higher long-term multilineage engraftment and greater self-renewing ability compared with that of AMD 3100. |
| Santiago 2015 | CasNa; AMD3100 | BALB/c | BALB/c | 8.5 Gy | 2 × 10^6^ PB MNCs | NA | 5 × 10^6^ BM MNCs from primary recipiens | Survival % for 20 weeks | The engraftment of the cells mobilized by CasNa was superior to cells mobilized by AMD3100. |
| Cao 2016 | AMD3100; BOP; AMD3100 + BOP | C57BL/6 | RFP^+^ mice | 10.5 Gy | 10-300 μl PB | PB cells from G-CSF-treated GFP^+^ cells | BM cells (1/10 femur) from primary recipients | Survival % for 120 days; Donor engraftment for 6-20 weeks | PB cells mobilized by AMD3100 + BOP resulted in greater survival and enhanced long-term multilineage engraftment compared with that of AMD3100 or G-CSF alone. |
| Lu 2016 | LECT2; G-CSF | B6.SJL (CD45.1^+^) | C57BL/6 (CD45.2^+^) | 9 Gy | 20 μl PB | 2 × 10^5^ BM cells from B6.SJL mice | NA | RU and CRU at 20 weeks | LECT2 mobilized more CRUs than G-CSF. |
| Karpova 2017 | POL5551; G-CSF | C57BL/6 (CD45.2^+^) | B6.SJL (CD45.1^+^) | 9.5-11 Gy | 2-8 μl PB | 2.5 × 10^5^ CD45.1/CD45.2 BM cells | NA | CRU at 12 weeks | 14-day POL5551 showed more potent mobilization compared with 5-day G-CSF as reflected in higher CRU frequency in limiting dilution transplantation assay. |
| Ogle 2017 | VPC01091; AMD3100; VPC01091 + AMD3100 | B6.SJL (CD45.1^+^) | C57BL/6 (CD45.2^+^) | 10.5 Gy | 8 × 10^6^ PB cells | 1 × 10^6^ CD45.1^+^ BM cells | NA | Donor chimerism at 6-16 weeks; Survival% for 8 weeks | HSPCs mobilized by VPC01091 + AMD3100 showed enhanced repopulation capacity and greater survival. |
| Wang 2017 | G-CSF + AMD3100; G-CSF + AMD3100 + Anti-Notch2 Ab | B6.BoyJ-Ly5.1 | C57BL/6-Ly5.2 | Lethally irradiated | 200 μl PB | 1 × 10^6^ Ly5.1 BM cells | NA | Donor chimerism for 3-13 weeks | Addition of Anti-Notch2 mobilized HSPCs with enhanced chimerism. |
| Hoggatt 2018 | tGROβ; AMD3100; G-CSF; AMD3100 + tGROβ | B6.BoyJ (CD45.1^+^) | C57BL/6 (CD45.2^+^) | 11 Gy | 1 × 10^6^ PBMCs | 5 × 10^5^ BM cells from BoyJ mice | 1 × 10^6^ BM cells from primary recipients | Donor chimerism and long-term multilineage reconstitution at 6 months | The tGROβ +AMD3100-mobilized grafts led to robust short- and long-term engraftment and was more competitive than G-CSF-mobilized grafts. |
| Karpova 2019 | G-CSF; tGROβ; Firategrast; CWHM-823; Firategrast + tGROβ; CWHM-823 + tGROβ | BALB/c (CD45.2^+^) | BALB/c (CD45.1^+^) | 9.5-11 Gy | 10 μl PB | 2.5 × 10^5^ CD45.2^+^ BM cells | 2.5 × 10^5^ BM cells from primary recipients | Donor chimerism at 20 weeks | The long-term engraftment and serial repopulating capacity of grafts mobilized by Firategrast/CWHM-823 + tGROβ are not inferior to G-CSF-mobilized grafts. |
| Liu 2019 | CASIN; AMD3100 | BoyJ (CD45.1^+^) | C57BL/6 (CD45.2^+^) | 11.75 Gy | 500 μl PB | NA | 5 × 10^6^ BM cells from primary recipients | Donor chimerism and long-term multilineage reconstitution at 6 months | CASIN-mobilized HSCs showed superior long-term reconstitution potential compared with AMD3100-mobilized HSCs. |
| Muller 2019 | LGB321 + AMD3100; AMD3100 | CD45.1^+^ mice | CD45.2^+^ mice | 8 Gy | 3 × 10^6^ PB cells | 1 × 10^6^ CD45.1/45.2 BM cells | NA | Donor chimerism at 18 weeks, multilineage reconstitution at 27 weeks | HSCs mobilized by LGB321 + AMD3100 exhibited a strong trend toward higher repopulating capacity compared to HSCs mobilized with AMD3100 alone. |
| Smith-Berdan 2019 | AMD3100 + Viagra; AMD3100 | B6.BoyJ or C57BL6 | C57BL6 or UCB-GFP | 10.25 Gy | Half or third of the total PB cells obtained | NA | BM cells from primary recipients | Donor chimerism for 8-20 weeks, multilineage reconstitution at 16 weeks | Viagra + AMD3100 increased the mobilization of functional, long-term engrafting multipotent HSCs compared with AMD3100 alone. |
| Szade 2019 | G-CSF; CoPP | GFP^-^ mice | UCB-GFP mice | 9 Gy | 5 × 10^6^ GFP^+^ PBMCs | 1 × 10^5^ GFP^-^ BM cells. | 1 × 10^7^ BM cells from primary recipients | Donor chimerism at 18 weeks | CoPP-mobilized cells produced higher chimerism than G-CSF-mobilized cells. |
| Fang 2021 | G-CSF; G-CSF + HF51116; G-CSF + AMD3100 | C57BL/6 × B6.SJL F1 (CD45.1/CD45.2) | C57BL/6 (CD45.2^+^) | 11 Gy | 1 × 10^6^ PBMNCs | 0.5 × 10^6^ CD45.1^+^ BM cells | BM cells from primary recipients | Donor chimerism for 1-6 months | G-CSF + HF51116 increased the mobilization of long-term repopulating and self-renewing HSCs compared with G-CSF alone and G-CSF + AMD3100. |
| Kaur 2021 | G-CSF; G-CSF + CSF1-Fc | B6.SJL CD45.1^+^ | C57BL/6 (CD45.2^+^) | 11.5 Gy | 20 μl PB | 2 × 10^5^ BM cells from UCB-GFP mice | NA | Donor chimerism for 8-16 weeks; multilineage reconstitution and RU at 16 weeks | HSPCs mobilized by CSF1-Fc + G-CSF exhibited greater reconstitution potential compared with cells mobilized by G-CSF alone. |

Abbreviations: BM, bone marrow; CRU, Competitive repopulating units; CRI, Competitive repopulating index; HSCs, hematopoietic stem cells; HSPCs, hematopoietic stem and progenitor cells; LTRC, long-term repopulating cells; NA, not applicable; MRA, marrow repopulating ability; PB, Peripheral blood; PBMCs, Peripheral blood mononuclear cells; RU, repopulating units.
